# Incidence, mortality, and cumulative risk of cancer in adolescents and young adults in Switzerland

**DOI:** 10.1007/s10654-026-01363-9

**Published:** 2026-02-21

**Authors:** Céline Bolliger, Eleftheria Michalopoulou, Christian Kreis, Matthias Lorez, Christina Schindera, Benjamin Kasenda, Claudia E. Kuehni, Daniela Dyntar, Katharina Roser, Ben D. Spycher

**Affiliations:** 1 Faculty of Health Sciences and Medicine, University of Lucerne, Lucerne, Switzerland; 2 Institute of Social and Preventive Medicine (ISPM), Faculty of Medicine, University of Bern, Bern, Switzerland; 3 National Institute for Cancer Epidemiology and Registration (NICER), Zurich, Switzerland; 4 Pediatric Hematology and Oncology, University Children’s Hospital Basel, Basel, Switzerland; 5 Department of Medical Oncology, University and University Hospital of Basel, Basel, Switzerland; 6 Division of Pediatric Hematology/Oncology, Department of Pediatrics, Inselspital, University Hospital Bern, Bern, Switzerland; 7 Childhood Cancer Registry, Institute of Social and Preventive Medicine, Faculty of Medicine, University of Bern, Bern, Switzerland

**Keywords:** Adolescent and young adult cancer, Cancer epidemiology, Incidence, Mortality, Cumulative risk, Switzerland

## Abstract

Cancers in adolescents and young adults (AYAs, aged 15–39 years) have distinct features and distribution of cancer types. This study investigated incidence, mortality, and cumulative risk of AYA cancers in Switzerland. Data on adolescent cancers (15–19 years; Swiss Childhood Cancer Registry) and young adult cancers (20–39 years; National Agency for Cancer Registration) were combined. We calculated age-standardized incidence and mortality rates, and accounted for incomplete regional coverage of cancer registration. We used joinpoint regression to investigate cancer incidence and mortality trends and estimate average annual percent changes. We included 44,048 cancers recorded between 1980 and 2019 and, after adjusting for incomplete coverage, we estimated a total of 69,901 cancers. Cancer incidence increased from 56.6 [95% confidence interval: 55.3, 58.0] per 100,000 person-years in 1980–1989 to 72.2 [71.1, 73.3] in 2010–2019, with a steeper increase for females. The most common cancers were carcinomas, mainly of the breast (9,629 cancers estimated), gastrointestinal tract (5,033) and thyroid (4,785), gonadal and related tumors (11,303), melanomas (9,695), and lymphomas (8,208). There were 9,865 AYA cancer deaths recorded over the study period. The age-standardized mortality rate declined from 13.3 [12.8, 13.7] per 100,000 person-years in 1980–1989 to 6.2 [5.9, 6.5] in 2010–2019, showing similar decreases in both sexes. This first comprehensive, nationwide study of AYA cancers in Switzerland showed increasing incidence and declining mortality, potentially due to changes in lifestyle or improved cancer registration and screening practices. Our results emphasize the need for ongoing surveillance and preventive strategies with a focus on AYAs.

**Supplementary Information:**

The online version contains supplementary material available at 10.1007/s10654-026-01363-9.

## Background

Cancer in adolescents and young adults (AYAs; commonly defined as the age group 15–39 years) has a distinct spectrum of cancer types, transitioning from cancers typically seen in childhood to those typical of adulthood [1, 2]. AYA cancers often exhibit distinct biological and molecular characteristics resulting in differences in clinical and treatment-related behavior [3].

AYA cancer survivors may suffer from physical and psychosocial late effects due to their cancer and its aggressive treatments [4, 5]. Because of their young age at diagnosis, AYA cancer survivors may experience disruptions to crucial developmental milestones such as education completion, financial independence from parents, career establishment, and family planning [6–10]. Despite significant advances in the understanding of cancer in AYAs, AYA cancers are commonly either grouped together with cancers in children or older adults or reported as a single age group, obscuring heterogeneity within the group [11].

Worldwide, more than one million new AYA cancers are diagnosed annually [12]. In Europe, approximately 66,000 AYA cancers are diagnosed each year [2]. Incidence differs between European countries [13] with the highest rates observed in south-western Europe and the lowest rates in Eastern Europe [2, 14]. Studies from Europe reported a decline in cancer mortality among AYAs over several decades [15, 16]. Still, cancer remains the leading cause of disease-related death in AYAs in high-income countries [12].

In Switzerland, there have been few epidemiological studies of cancer in AYAs, and these focused either on a divergent age group and time period, e.g. young adults aged 20–44 years between 1951–1992 [17, 18], or only on specific cancer types such as breast [19] and testicular cancer [20]. A comprehensive epidemiological study covering the whole age range and all cancers in AYAs in Switzerland is lacking.

This study combines all available cancer registry data in Switzerland to investigate incidence, mortality, and cumulative risk of AYA cancer during 1980–2019.

## Methods

### Patients and population at risk

The study population comprised AYAs aged 15–39 years residing in Switzerland between 1980 and 2019 (study period). We obtained data on primary cancers diagnosed in this population during the study period in adolescents (age 15–19 years) and in young adults (age 20–39 years) from the Swiss Childhood Cancer Registry (ChCR), and the National Agency for Cancer Registration (NACR), respectively. To estimate cumulative risk, we also obtained primary cancers in children (age 0-14 years) from the ChCR. By selecting the source of registry data strictly based on age at time of diagnosis, we precluded any duplicate records of adolescent cancers recorded both in the ChCR and cantonal registries. Primary cancers were determined according to the International Rules for Mutliple Primary Cancers [21]. We did not obtain any data of cancers diagnosed in patients who had objected to their registration, but were informed by our data providers that these were few (less than 20 patients).

Until it became compulsory under federal law in January 2020, cancer registration in Switzerland was under the jurisdiction of the cantons. The 13 existing cantonal cancer registries (CCRs) collect data on cancers diagnosed in the population residing in Switzerland and share these data with the NACR, which maintains a standardized dataset for central storage and dissemination. The first CCRs were established in predominately French-speaking cantons during the 1970s, but half of the 26 cantons, including a majority of predominately German-speaking cantons, only started cancer registration over the last two decades (see Table S1 of the supplementary material). Consequently, registration coverage has been incomplete in the past. For children (0–14 years, needed for estimating cumulative risks) and adolescents (15–19 years), we obtained data from the ChCR. The ChCR was established in 1976 and had nationwide coverage for cancers at ages 0–15 years during the entire study period. It also registered cancers at age 16–20 years, but this relied heavily on data exchanges with existing CCRs, resulting again in notable regional differences.

The proportion of the population residing in Switzerland covered by the CCRs increased from 41 to 88% during 1980–2019. A study conducted in 2017 evaluating completeness of case ascertainment within 10 CCRs reported high levels of completeness within the regions covered [22].

From the Federal Statistical Office (FSO), we obtained nationwide individual-level data on death and principal cause of death as well as data on the Swiss resident population at year-end by sex, age, calendar year, and canton of residence, covering the study period.

### Cancer categories

The CCRs and the ChCR coded primary cancers according to the International Classification of Diseases for Oncology (ICD-O, version 3.0 for diagnoses before 2012 and version 3.1 thereafter) [23]. We recoded cancers according to the classification system for AYAs proposed by Barr et al. [24], excluding in situ cancers (Barr’s category A) and non-melanotic skin cancers (ICD-10 C44, Barr’s category 9.5). Causes of death were coded according to the International Statistical Classification of Diseases and Related Health Problems (ICD, version 8 for deaths before 1995 [25] and version 10 [26] thereafter). Hence, we could not recode them according to Barr’s AYA cancer classification system, which was based on ICD-O. Following the 2021 Swiss Cancer Report [27], we included deaths due to the following cancers: 1. Lip, oral cavity, and pharynx, 2. Oesophagus, 3. Stomach, 4. Colon, 5. Rectosigmoid junction and rectum, 6. Liver and intrahepatic bile ducts, 7. Biliary tract, 8. Pancreas, 9. Larynx, 10. Trachea, bronchus, and lung, 11. Pleura mesothelioma, 12. Melanoma of skin, 13. Breast, 14. Cervix uteri, 15. Corpus uteri, 16. Ovary, 17. Prostate, 18. Testis, 19. Kidney, 20. Bladder, 21. Brain and central nervous system (CNS), 22. Thyroid, 23. Hodgkin lymphoma, 24. Non-Hodgkin lymphoma, 25. Multiple myeloma, 26. Leukemia, 27. Other and unspecified malignant neoplasms. Moreover, we included deaths due to benign or uncertain neoplasms of brain and CNS (ICD-10: D18, D32, D33, D43, D44), resulting in a total of 28 cancer death categories.

### Statistical analysis

To adjust for incomplete registration coverage in Switzerland, we extrapolated the observed number of primary cancers to estimate the number of cancers that would have been observed under nationwide coverage. Extrapolation weights were calculated as the ratio of the resident population in Switzerland over the population covered by the existing CCRs in a given year stratified by language region (predominately German-speaking cantons, predominately French- or Italian-speaking cantons referred to here as German-speaking and French/Italian-speaking cantons, respectively), sex (male, female), and age group (15–19, 20–24, 25–29, 30–34, 35–39 years). The overall number of primary cancers for a calendar period was estimated as the weighted sum of stratum-specific observed cases during that period. As the cause of death statistics have nationwide coverage, no weighting was required for cancer deaths. However, we applied sex- and cancer-death-category-specific correction factors as calculated by Lutz et al. [28] to cancer deaths before 1995. This correction aimed to avoid the overestimation of cancer mortality rates during 1980–1994 due to a prioritization scheme used by the FSO at that time to assign causes of death that was subsequently changed [28]. The correction factors were not applied to cancer death categories not listed in Lutz et al. (i.e. to 2. Oesophagus, 6. Liver and intrahepatic bile ducts, 7. Biliary tract, 9. Larynx, 11. Pleura mesothelioma, 19. Kidney, 23. Hodgkin lymphoma, 24. Non-Hodgkin lymphoma, 25. Multiple myeloma, 26. Leukemia, 27. Other and unspecified malignant neoplasms, 28. Benign or uncertain neoplasms of brain and CNS).

We calculated crude incidence (mortality) rates per 100,000 person years for each cancer category (cancer death category) and 10-year calendar period stratified by sex and age group at diagnosis. Crude rates were calculated by dividing the aggregated estimated number of cancers (observed number of cancer deaths) for a given calendar period and stratum by the corresponding sum of the mid-year population during that period. The mid-year population for a given year was calculated as the mean of the end-year populations of that and the preceding year. We also calculated directly age-standardized incidence (mortality) rates per 100,000 person years, using the 1976 European Standard Population [29] (as well as the updated 2013 version; Tables S2 and S3 of the supplementary material). We calculated 95% confidence intervals (CIs) for the age-standardized rates as suggested by Fay and Feuer [30], incorporating the extrapolation weights in the variance estimation [31]. For the crude mortality rates, which are simply scaled counts, we calculated 95% CIs based on the Poisson distribution [32]. Throughout this manuscript, 95% CIs are displayed in square brackets after the estimates (e.g. rates, average annual percent changes).

Age-standardized incidence (mortality) trends during 1980–2019 were examined using the Joinpoint Trend Analysis Software (version 4.9.1.0) by the National Cancer Institute (NCI) of the National Institutes of Health (NIH) [33, 34]. We report the average annual percent change (AAPC) for the entire study period, as calculated by the Joinpoint software, selecting the parametric method for calculating 95% CIs. We identified breaks in trends using the grid search method based on permutation tests with default settings, and a maximum of 7 joinpoints since we had data covering 40 years. We assumed heteroscedastic and uncorrelated errors.

Based on incidence and mortality data from 2015–2019 (including children < 15 years), we estimated the cumulative risk of developing cancer at any age before 40 years and the cumulative risk for persons still cancer-free on their 15th birthday to develop cancer before age 40 years (during the AYA age window). We followed the methodology by Fay et al. [30], which assumes death as the only competing risk and that patients with cancer have the same risk of non-cancer death as the general population.

To assess the potential impact on incidence trends of the extrapolation method used to adjust incidence estimates for incomplete registry coverage, we conducted a sensitivity analysis by restricting our main trend analysis to a total of seven cantons with continuous cancer registration in Switzerland throughout the study period. These cantons encompassed on average 41.6% of the national AYA population during this period. We then compared the resulting incidence and mortality trends to those from our main analyses.

## Results

### Study population

We obtained data on 44,081 cancers satisfying our inclusion criteria from the CCRs and the ChCR. After excluding 4 primary adolescent cancers with missing information on canton of residence at diagnosis, and 29 primary young adult cancers with uncertain date of diagnosis, we included 44,048 primary cancers diagnosed during 1980–2019 (Table 1). Out of those, 23,832 (54%) cancers were diagnosed in females.

**Table 1:** Observed number of primary cancers (n) and proportion of the Swiss population covered by the existing cantonal cancer registries (coverage; %) for different time periods (1980–1989, 1990–1999, 2000–2009, 2010–2019) by language region, sex, and age at diagnosis

Population characteristics	Total n	Coverage (%)	Period							
			1980–1989		1990–1999		2000–2009		2010–2019	
			n	(%)	n	(%)	n	(%)	n	(%)
All cancers	44′048	63.1	6′846	49	9′497	56	10′850	61	16′855	84
Language region										
German /Romansh	27′269	55.7	4′448	45	5′755	48	5′862	49	11′204	80
French/Italian	16′779	81.1	2′398	61	3′742	77	4′988	91	5′651	93
Sex										
Female	23′832	63.2	3′561	50	4′908	57	5′993	62	9′370	84
Male	20′216	63.0	3′285	49	4′589	56	4′857	61	7′485	84
Age at diagnosis (years)										
15–19	2′529	65.4	427	55	536	63	626	66	940	80
20–24	4′597	61.5	813	47	945	55	1′106	60	1′733	84
25–29	7′596	62.9	1′203	48	1′741	55	1′723	61	2′929	85
30–34	12′001	63.0	1′721	48	2′674	55	2′869	61	4′737	85
35–39	17′325	62.8	2′682	49	3′601	55	4′526	60	6′516	85

On average, the existing Swiss registries covered 63% of the national AYA population during the study period. Registration coverage was higher in the French/Italian-speaking cantons as compared to the German-speaking cantons (81.1% vs. 55.7%) and increased over the study period from 49.4% during 1980–1989 to 84.1% during 2010–2019 (Table 1).

### Incidence

We estimated a total of 69,901 cancers for the entire country during 1980–2019 (Table 2). Females accounted for more than half of the estimated cancers (54% of the total). Almost 2/3 were diagnosed at the age ≥ 30 years (30–34 years: 27%, 35–39: 39%). Carcinomas (cancer category 9) was the most common cancer category (38.5%) followed by 7. Gonadal and related tumors (16.2%), 8. Melanoma (13.9%), and 2. Lymphomas (11.7%).

**Table 2:** Estimated number of primary cancers (n), age-standardized incidence (per 100′000 person years) and average annual percent change (AAPC) of cancer in AYAs in Switzerland for different time periods between 1980 and 2019 by language region, sex, age at diagnosis, and cancer category

Characteristics	Estimated number of primary cancers (n)					
	Total^1^ (%)		1980–1989	1990–1999	2000–2009	2010–2019
All cancers	69′900.8	(100.0)	14′046.4	17′130.6	17′664.6	19′874.0
Language region						
German	49′305.9	(70.5)	10′118.1	59′965.6	59′914.2	70′121.7
French/Italian	20′594.9	(29.5)	3′928.3	24′221.2	27′308.3	30′474.7
Sex						
Female	37′681.1	(53.9)	7′250.6	8′829.6	9′698.3	11′029.2
Male	32′219.7	(46.1)	6′795.8	8′300.9	7′966.3	8′844.8
Age at diagnosis (years)						
15–19	3′873.2	(5.5)	777.3	863.8	974.3	1′175.6
20–24	7′456.1	(10.7)	1′715.1	1′733.2	1′841.7	2′057.0
25–29	12′059.7	(17.3)	2′489.1	3′174.6	2′754.9	3′446.7
30–34	19′052.9	(27.3)	3′581.7	4′870.7	4′662.3	5′547.1
35–39	27′459.0	(39.3)	5′483.3	6′488.4	7′431.5	7′647.6
Cancer category^2,3^						
1. Leukemias and related disorders	3′278.2	(4.7)	632.5	741.5	892.9	951.5
1.1 Acute lymphoblastic leukemia	650.4	(0.9)	148.1	155.8	158.8	179.0
1.2 Acute myeloid leukemia	1′051.8	(1.5)	249.3	278.0	280.0	255.4
1.3 Chronic myeloid leukemia	569.1	(0.8)	115.9	152.9	161.9	143.7
1.6 Essential thrombocythemia	249.6	(0.4)	12.2	28.0	58.0	118.8
2. Lymphomas	8′208.2	(11.7)	1′649.4	2′162.1	2′098.1	2′239.3
2.1 Non-Hodgkin lymphomas	3′384.7	(4.9)	607.4	1′020.6	894.3	863.4
2.1.2 Burkitt	252.1	(0.5)	47.6	77.9	72.9	55.2
2.1.3 Diffuse large B-cell (DLBCL)	1′312.4	(2.4)	221.7	477.6	358.7	284.7
2.1.5 Anaplastic T-cell and null-cell excluding NK/T-cell	478.2	(0.9)	70.7	111.2	135.3	145.5
2.1.6 Follicular	420.9	(0.8)	69.1	98.5	117.6	125.4
2.2 Hodgkin lymphoma	4′243.0	(6.1)	846.7	973.0	1′098.3	1′243.6
2.2.2 Hodgkin classic, other	4′059.9	(7.4)	846.7	964.7	1′040.1	1′153.3
3. CNS and other intracranial and intraspinal neoplasms	5′410.5	(7.7)	944.3	1′064.6	1′332.2	1′789.5
3.1 Astroglial and related neoplasms	2′825.6	(4.1)	585.0	649.1	722.8	809.6
3.1.1 Oligodendriogliomas	564.2	(1.0)	84.1	115.0	165.6	176.2
3.1.2 Glioblastomas/gliofibromas	471.1	(0.9)	75.0	106.4	137.4	137.6
3.1.3 Ependymomas	381.4	(0.7)	61.0	47.0	92.0	147.1
3.4 Neuronal and mixed neuronal-glial neoplasms	259.3	(0.4)	31.2	30.2	74.7	102.4
3.4.1 Neuronal and mixed neuronal-glial, benign/borderline	252.6	(0.5)	28.9	25.8	73.6	102.4
3.5 Meningiomas	1′084.4	(1.6)	119.1	187.9	249.9	426.5
3.5.1 Meningioma, benign/borderline	1′043.1	(1.9)	107.7	173.7	243.6	416.0
3.8 Pituitary neoplasms	662.4	(1.0)	85.3	91.4	152.5	268.8
3.8.1 Pituitary, benign/borderline	640.5	(1.2)	68.2	85.3	152.5	266.5
4. Sarcomas	2′915.2	(4.2)	642.9	754.0	711.4	791.1
4.1 Osteosarcoma	347.8	(0.5)	78.0	97.0	86.7	88.5
4.2 Chondrosarcoma	294.5	(0.4)	46.1	79.7	72.6	86.6
4.3 Ewing family of tumors	300.0	(0.4)	66.4	66.0	56.3	99.1
4.4 Fibromatous neoplasms	589.9	(0.9)	143.3	203.5	133.1	128.7
4.5 Liposarcoma	259.3	(0.4)	60.3	48.0	80.2	67.5
5. Blood and lymphatic vessel tumor	1′073.7	(1.5)	303.7	539.2	192.4	134.1
5.2 Malignant blood and lymphatic vessel tumors, all sites	899.6	(1.3)	270.6	517.4	133.2	81.2
5.2.1 Kaposi sarcoma	764.0	(1.4)	233.7	486.3	101.3	44.2
6. Nerve sheath tumors	615.0	(0.9)	75.9	133.6	135.7	228.4
6.1 Benign, CNS	499.3	(0.7)	57.4	99.0	109.1	193.0
6.1.1 Neurilemmoma	472.7	(0.9)	52.8	92.4	102.8	183.9
7. Gonadal and related tumors	11′302.6	(16.2)	2′598.9	2′896.4	2′765.9	3′000.6
7.1 Testis	9′781.1	(14.1)	2′171.7	2′461.4	2′443.1	2′634.0
7.1.1 Germ cell and trophoblastic	9′748.9	(17.7)	2′167.8	2′451.0	2′431.6	2′626.9
7.2 Ovary	1′110.4	(1.6)	307.8	328.9	232.5	262.1
7.2.1 Germ cell and trophoblastic	288.0	(0.5)	67.7	76.4	49.4	88.1
7.2.2 Non-germ cell	822.4	(1.5)	240.1	252.5	183.1	174.0
7.4 Germ cell and trophoblastic excluding CNS, ovary, testis	323.5	(0.5)	104.3	86.3	72.7	73.9
8. Melanoma, malignant	9′695.0	(13.9)	1′506.6	2′503.5	2′871.7	2′609.7
8.1 Superficial spreading/low cumulative sun damage melanoma	5′792.9	(8.3)	742.1	1′493.6	1′729.0	1′630.7
8.2 Nodular melanoma	864.2	(1.2)	273.6	282.2	234.1	135.0
9. Carcinomas	26′901.5	(38.5)	5′530.2	6′198.0	6′543.6	8′028.7
9.1 Thyroid carcinoma	4′784.9	(6.9)	572.5	909.4	1′239.5	1′746.0
9.1.3 Papillary	2′879.1	(5.2)	238.2	394.8	790.5	1′173.0
9.1.4 Follicular	606.6	(1.1)	82.4	157.0	180.9	176.8
9.1.5 Papillary with follicular variant	1′053.3	(1.9)	179.0	274.5	218.1	344.9
9.2 Other carcinoma of head and neck	1′368.9	(2.0)	339.6	376.6	334.2	327.7
9.2.2 Oral cavity, lip, and pharynx	707.1	(1.3)	199.3	178.3	169.7	168.0
9.2.3 Salivary gland	249.3	(0.5)	31.1	83.8	57.4	69.7
9.3 Carcinoma of gastrointestinal tract	5′033.4	(7.2)	957.4	1′148.7	1′090.6	1′647.2
9.3.2 Carcinoma of stomach	888.7	(1.6)	225.4	252.5	211.8	212.9
9.3.4 Carcinoma of colon	2′074.1	(3.8)	330.5	374.2	404.5	809.0
9.3.5 Carcinoma of rectum	935.0	(1.7)	186.3	223.5	200.5	296.3
9.3.7 Carcinoma of liver and intrahepatic bile ducts (IBD)	270.0	(0.5)	48.9	73.2	73.7	72.9
9.3.9 Carcinoma of pancreas	350.4	(0.6)	61.7	90.7	72.6	113.5
9.4 Carcinoma of lung, bronchus, and trachea	1′227.2	(1.8)	358.4	355.7	264.7	282.9
9.4.2 Non-small cell carcinoma	1′110.6	(2.0)	300.9	309.1	241.2	275.9
9.6 Carcinoma of breast	9′629.3	(13.8)	1′984.5	2′030.2	2′467.9	2′896.3
9.6.1 Breast, infiltrating duct	8′045.7	(14.6)	1′346.3	1′663.9	2′122.8	2′576.5
9.6.2 Breast, adenocarcinoma	476.5	(0.9)	310.1	59.4	67.3	85.1
9.6.3 Breast, lobular	437.1	(0.8)	116.2	127.4	126.4	84.1
9.6.5 Breast, medullary	253.2	(0.5)	84.8	67.2	69.3	47.4
9.7 Carcinoma of genital sites excluding ovary and testis	3′405.1	(4.9)	970.4	1′012.0	809.6	729.0
9.7.1 Carcinoma of uterine cervix	2′791.6	(5.1)	844.3	859.5	611.8	586.2
9.7.2 Corpus uteri	407.3	(0.7)	72.2	98.1	126.1	108.4
9.8 Carcinoma of urinary tract	1′009.7	(1.5)	234.4	231.2	242.2	288.2
9.8.1 Carcinoma of kidney	701.9	(1.3)	108.0	139.7	185.8	233.6
9.8.2 Carcinoma of bladder	264.2	(0.5)	110.7	81.6	45.8	45.1
10. Miscellaneous specified neoplasms	236.5	(0.3)	76.0	65.4	71.4	39.0
11. Unspecified malignant neoplasms except CNS	264.5	(0.4)	86.0	72.3	49.5	62.1

Characteristics	Age-standardized incidence rate per 100'000 person years					AAPC [95% CI]
	Total^1^	1980–1989	1990–1999	2000–2009	2010–2019	1980–2019
All cancers	66.0	56.6	63.7	66.3	72.2	1.0 [0.7, 1.3]
Language region						
German	65.6	56.6	64.1	65.1	71.6	1.0 [0.5, 1.4]
French/Italian	66.9	56.7	62.7	69.2	73.6	0.9 [0.7, 1.0]
Sex						
Female	71.1	59.0	65.9	71.9	80.3	1.0 [0.8, 1.1]
Male	60.9	54.4	61.6	60.7	64.1	0.9 [0.4, 1.4]
Age at diagnosis (years)						
15–19	21.9	15.8	21.3	22.5	26.8	1.6 [1.2, 1.9]
20–24	39.4	34.0	37.8	42.0	42.0	0.6 [0.2, 0.9]
25–29	58.6	50.8	58.0	58.2	63.0	0.6 [0.4, 0.8]
30–34	86.9	72.5	83.3	86.5	96.5	0.5 [-08., 1.8]
35–39	123.0	110.0	118.0	122.5	132.6	0.6 [0.5, 0.8]
Cancer category^2,3^						
1. Leukemias and related disorders	3.2	2.6	2.9	3.5	3.6	1.2 [0.7, 1.6]
1.1 Acute lymphoblastic leukemia	0.7	0.6	0.7	0.7	0.7	0.9 [0.1, 1.7]
1.2 Acute myeloid leukemia	1.0	1.0	1.1	1.1	1.0	−0.1 [−0.7, 0.5]
1.3 Chronic myeloid leukemia	0.5	0.5	0.6	0.6	0.5	0.3 [−0.7, 1.2]
1.6 Essential thrombocythemia	0.2	0.1	0.1	0.2	0.4	4.8 [3.4, 6.3]
2. Lymphomas	8.0	6.7	8.3	8.3	8.4	0.6 [0.2, 0.9]
2.1 Non-Hodgkin lymphomas	3.2	2.5	3.8	3.4	3.2	1.5 [0.3, 2.7]
2.1.2 Burkitt	0.2	0.2	0.3	0.3	0.2	−0.6 [−2.0, 0.8]
2.1.3 Diffuse large B-cell (DLBCL)	1.3	0.9	1.8	1.4	1.0	1.9 [0.1, 3.6]
2.1.5 Anaplastic T-cell and null-cell excluding NK/T-cell	0.5	0.3	0.4	0.5	0.5	1.1 [0.1, 2.2]
2.1.6 Follicular	0.4	0.3	0.4	0.4	0.5	0.9 [−0.1, 2.1]
2.2 Hodgkin lymphoma	4.2	3.4	3.9	4.5	4.8	1.1 [0.7, 1.4]
2.2.2 Hodgkin classic, other	4.0	3.4	3.8	4.3	4.4	0.8 [0.4, 1.2]
3. CNS and other intracranial and intraspinal neoplasms	5.2	3.8	4.1	5.1	6.6	1.9 [1.6, 2.3]
3.1 Astroglial and related neoplasms	2.7	2.4	2.5	2.8	3.0	0.8 [0.3, 1.2]
3.1.1 Oligodendriogliomas	0.5	0.3	0.4	0.6	0.6	1.2 [−0.1, 2.6]
3.1.2 Glioblastomas/gliofibromas	0.4	0.3	0.4	0.5	0.5	1.3 [0.2, 2.4]
3.1.3 Ependymomas	0.4	0.3	0.2	0.4	0.6	0.0 [−3.3, 3.5]
3.4 Neuronal and mixed neuronal-glial neoplasms	0.3	0.1	0.1	0.3	0.4	3.4 [2.1, 4.7]
3.4.1 Neuronal and mixed neuronal-glial, benign/borderline	0.3	0.1	0.1	0.3	0.4	3.6 [2.3, 5.0]
3.5 Meningiomas	1.0	0.5	0.7	0.9	1.5	3.5 [2.8, 4.3]
3.5.1 Meningioma, benign/borderline	1.0	0.4	0.6	0.9	1.5	3.7 [2.9, 4.4]
3.8 Pituitary neoplasms	0.6	0.3	0.3	0.6	1.0	4.0 [2.9, 5.1]
3.8.1 Pituitary, benign/borderline	0.6	0.3	0.3	0.6	1.0	4.5 [3.4, 5.6]
4. Sarcomas	2.9	2.6	2.9	2.8	3.0	0.3 [-0.2, 0.8]
4.1 Osteosarcoma	0.4	0.3	0.4	0.4	0.4	0.2 [−1.0, 1.4]
4.2 Chondrosarcoma	0.3	0.2	0.3	0.3	0.3	0.6 [-0.9, 2.1]
4.3 Ewing family of tumors	0.3	0.3	0.3	0.2	0.4	1.0 [−0.3, 2.2]
4.4 Fibromatous neoplasms	0.6	0.6	0.8	0.5	0.5	−0.9 [−2.0, 0.2]
4.5 Liposarcoma	0.3	0.2	0.2	0.3	0.2	0.2 [−1.2, 1.7]
5. Blood and lymphatic vessel tumor	1.0	1.2	1.9	0.7	0.5	3.5 [−1.9, 9.1]
5.2 Malignant blood and lymphatic vessel tumors, all sites	0.8	1.1	1.9	0.5	0.3	1.6 [−3.8, 7.2]
5.2.1 Kaposi sarcoma	0.7	0.9	1.7	0.4	0.2	−1.1 [−9.7, 8.4]
6. Nerve sheath tumors	0.6	0.3	0.5	0.5	0.8	2.7 [1.6, 3.7]
6.1 Benign, CNS	0.5	0.2	0.4	0.4	0.7	2.9 [1.8, 4.0]
6.1.1 Neurilemmoma	0.4	0.2	0.4	0.4	0.7	2.8 [1.6, 4.1]
7. Gonadal and related tumors	10.8	10.5	10.8	10.7	11.0	0.5 [−0.1, 1.1]
7.1 Testis	9.3	8.8	9.1	9.4	9.6	0.3 [0.0, 0.6]
7.1.1 Germ cell and trophoblastic	9.3	8.7	9.1	9.4	9.6	0.3 [0.0, 0.6]
7.2 Ovary	1.1	1.2	1.2	0.9	1.0	−1.0 [−1.7, −0.3]
7.2.1 Germ cell and trophoblastic	0.3	0.3	0.3	0.2	0.4	0.7 [−0.7, 2.1]
7.2.2 Non-germ cell	0.8	1.0	0.9	0.7	0.6	−1.6 [−2.4, −0.8]
7.4 Germ cell and trophoblastic excluding CNS, ovary, testis	0.3	0.4	0.3	0.3	0.3	−1.5 [−2.5, −0.4]
8. Melanoma, malignant	9.1	6.1	9.3	10.8	9.3	1.3 [0.6, 1.9]
8.1 Superficial spreading/low cumulative sun damage melanoma	5.4	3.0	5.5	6.5	5.8	1.8 [0.9, 2.6]
8.2 Nodular melanoma	0.8	1.1	1.1	0.9	0.5	−2.8 [−4.5, −1.1]
9. Carcinomas	24.8	22.3	22.5	23.5	28.6	0.8 [0.5, 1.1]
9.1 Thyroid carcinoma	4.5	2.3	3.4	4.7	6.3	2.9 [2.1, 3.6]
9.1.3 Papillary	2.7	1.0	1.5	3.0	4.2	4.9 [4.3, 5.5]
9.1.4 Follicular	0.6	0.3	0.6	0.7	0.7	0.7 [−0.9, 2.4]
9.1.5 Papillary with follicular variant	1.0	0.7	1.0	0.8	1.3	−0.1 [−3.6, 3.6]
9.2 Other carcinoma of head and neck	1.3	1.4	1.4	1.2	1.2	−0.7 [−1.4, 0.0]
9.2.2 Oral cavity, lip, and pharynx	0.7	0.8	0.7	0.6	0.6	−1.0 [−2.0, 0.1]
9.2.3 Salivary gland	0.2	0.1	0.3	0.2	0.3	0.5 [−1.3, 2.3]
9.3 Carcinoma of gastrointestinal tract	4.7	3.9	4.2	4.0	6.0	1.4 [0.8, 2.1]
9.3.2 Carcinoma of stomach	0.8	0.9	0.9	0.8	0.8	−1.0 [−1.7, −0.3]
9.3.4 Carcinoma of colon	2.0	1.3	1.4	1.5	3.0	2.8 [1.6, 4.1]
9.3.5 Carcinoma of rectum	0.9	0.8	0.8	0.7	1.1	1.0 [0.2, 1.9]
9.3.7 Carcinoma of liver and intrahepatic bile ducts (IBD)	0.3	0.2	0.3	0.3	0.3	0.2 [−1.1, 1.5]
9.3.9 Carcinoma of pancreas	0.3	0.3	0.3	0.3	0.4	1.3 [−0.1, 2.7]
9.4 Carcinoma of lung, bronchus, and trachea	1.1	1.4	1.3	1.0	1.0	−1.2 [−1.9, −0.6]
9.4.2 Non-small cell carcinoma	1.0	1.2	1.1	0.9	1.0	−0.8 [−1.6, −0.1]
9.6 Carcinoma of breast	8.8	8.0	7.3	8.6	10.1	0.7 [0.1, 1.3]
9.6.1 Breast, infiltrating duct	7.3	5.4	6.0	7.4	9.0	3.0 [1.3, 4.8]
9.6.2 Breast, adenocarcinoma	0.4	1.3	0.2	0.2	0.3	−6.2 [−8.6, −3.8]
9.6.3 Breast, lobular	0.4	0.5	0.5	0.4	0.3	−1.4 [−2.4, −0.3]
9.6.5 Breast, medullary	0.2	0.3	0.2	0.2	0.2	−1.8 [−3.2, −0.4]
9.7 Carcinoma of genital sites excluding ovary and testis	3.1	3.9	3.6	2.9	2.6	−1.6 [−1.9, −1.2]
9.7.1 Carcinoma of uterine cervix	2.6	3.4	3.1	2.2	2.1	−1.9 [−2.3, −1.5]
9.7.2 Corpus uteri	0.4	0.3	0.4	0.4	0.4	0.6 [−0.4, 1.6]
9.8 Carcinoma of urinary tract	0.9	0.9	0.8	0.9	1.0	0.2 [−0.6, 1.0]
9.8.1 Carcinoma of kidney	0.7	0.4	0.5	0.7	0.8	1.9 [0.9, 2.9]
9.8.2 Carcinoma of bladder	0.2	0.5	0.3	0.2	0.2	−3.3 [−4.6, −2.1]
10. Miscellaneous specified neoplasms	0.2	0.3	0.3	0.3	0.2	−2.0 [−3.2, −0.8]
11. Unspecified malignant neoplasms except CNS	0.3	0.4	0.3	0.2	0.2	−1.2 [−2.5, 0.1]

*AAPC* average annual percent change; *CNS* central nervous system

^1^ The total corresponds to the whole of the study period, 1980–2019

^2^ Cancer categories according to Barr et al.[24]

^3^ Rare cancer categories (n < 249), 4th and lower level sub-categories (e.g. 3.1.1.1–3.1.1.2), and 2nd and lower level sub-categories of "Other" cancers (e.g. 1.9 Other and unspecified leukemias, 3.10 Other and unspecified CNS) are not reported

Overall, age-standardized incidence for all cancers increased over time, from 56.6 cancers per 100,000 person years in 1980–1989 to 72.2 in 2010–2019 (AAPC: 1.0% [0.7, 1.3]) (Table 2). Age-standardized incidence rates were higher in the French/Italian- than in the German-speaking cantons, especially after 2000. Age-standardized incidence was higher in females (71.1 [70.2, 72.1] during 1980–2019) compared to males (60.9 [60.0, 61.7) (sex-specific tables analogous to Table 1: Table S4 and S5), with an average increase of about 1.0% per year for both sexes (AAPC males: 0.9% [0.4, 1.4], AAPC females: 1.0% [0.8, 1.1]). Overall incidence rates were higher for older age groups: 132.6 [129.4, 135.9] vs. 26.8 [25.1, 28.6] in age groups 35–39 and 15–19 years, respectively, during 2010–2019. Cancer categories with the highest age-standardized incidence rates during 1980–2019 were testis (9.3 [9.1, 9.6]), malignant melanoma (referred to here simply as melanoma, 9.1 [8.9, 9.3]), carcinoma of breast (8.8 [8.5, 9.0]), thyroid carcinoma (4.5, [4.4, 4.7]), carcinoma of gastrointestinal tract (4.7 [4.5, 4.9]), non-Hodgkin lymphomas (3.2 [3.1, 3.4]), and carcinoma of genital sites excluding ovary and testis (3.1 [3.0, 3.3]).

**Fig. 1:**
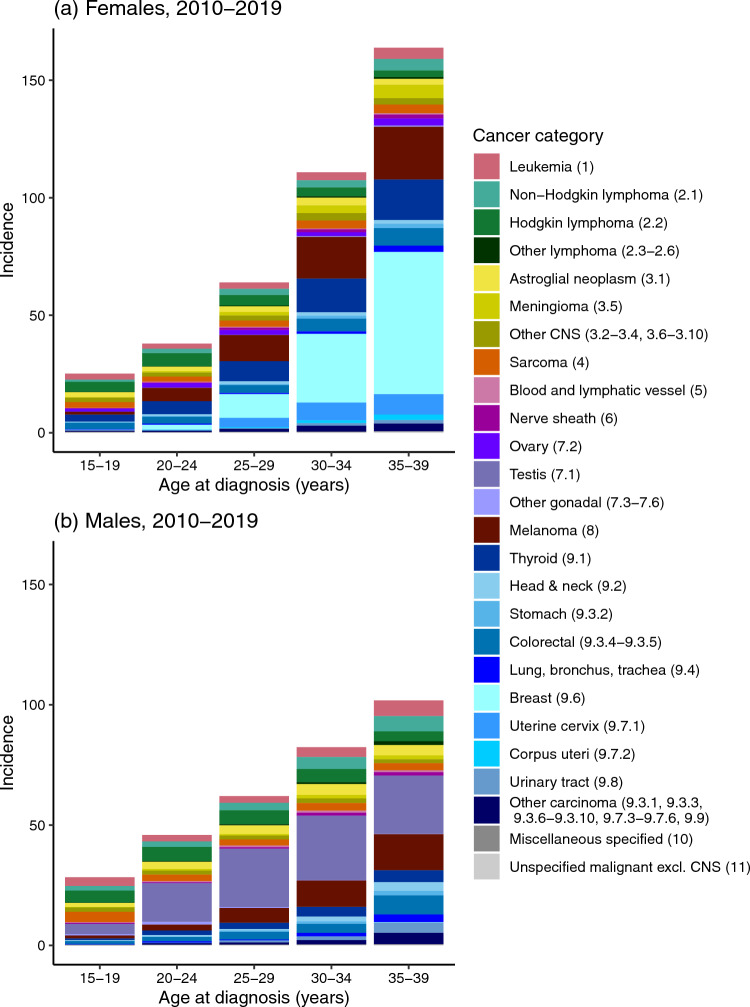
Age-specific incidence per 100′000 person years by cancer category (number according to Barr et al. [24]) in females (**a**) and males (**b**) in Switzerland for the time period 2010–2019

Figure 1 displays age-specific cancer incidence and the distribution of cancer types in females (a) and in males (b) during 2010–2019. For both sexes, the adolescent period (15–19 years) was dominated by cancers typically occurring during childhood, such as leukemias, lymphomas, CNS neoplasms, and sarcomas. With increasing age carcinomas became dominant. The most frequent cancer in females was breast carcinoma, with incidence increasing steadily with age (15–19 years: 0.2 [0.0, 0.5], 20–24: 2.0 [1.4, 2.7], 25–29: 10.1 [8.9, 11.5], 30–34: 29.2 [27.1, 31.4], 35–39: 60.5 [57.5, 63.7]). The most frequent cancer in males was testicular cancer, with a relatively high incidence across all age groups peaking at age 30–34 years (15–19: 4.2 [3.3, 5.3], 20–24: 16.0 [14.4, 17.9], 25–29: 24.1 [22.2, 26.2], 30–34: 26.7 [24.6, 28.8], 35–39: 24.1 [22.2, 26.1]). Melanoma, CNS and other intracranial and intraspinal neoplasms, and carcinoma of the colon and rectum frequently occurred in both sexes with incidence increasing with age. The incidence of thyroid carcinoma was markedly higher in females than in males and highest for the oldest age group (females 35–39 years: 17.3 [15.7, 19.1], males 35–39 years: 5.1 [4.2, 6.0]). The incidence of haematological cancers was generally higher in males than in females (leukemia, 15–19 years, males 3.7 [2.8, 4.7] vs. females 2.5 [1.8, 3.4], 35–39 years: 6.5 [5.6, 7.6] vs. 4.8 [4.0, 5.8]; non-Hodgkin lymphoma 15–19 years: 1.8 [1.3, 2.6] vs. 0.9 [0.5, 1.5], 35–39 years: 6.3 [5.4, 7.4] vs. 4.8 [4.0, 5.8]; and Hodgkin lymphoma 15–19 years: 5.0 [4.0, 6.2] vs. 4.3 [3.4, 5.5], 35-39 years: 4.2 [3.4, 5.0] vs. 2.9 [2.6, 3.7]).

### Cumulative risk

The cumulative risk of developing a first primary cancer before the age of 40 years was estimated at 2.0% (2.2% in females and 1.8% in males). The estimated cumulative risk for an individual free of cancer on their 15th birthday to be diagnosed with cancer before turning 40 years (i.e. a diagnosis during the AYA period) was 1.7% (1.9% in females and 1.5% in males).

### Mortality

In total 9,865 deaths due to cancer were recorded among Swiss resident AYAs during 1980–2019, of which 66% occured above the age of 30 years. Age-standardized mortality for all cancers decreased from 13.3 cancer deaths per 100,000 person years in 1980–1989 to 6.2 in 2010–2019 (Table 3). Age-standardized mortality was slightly higher in females compared to males except during the earliest calendar period. The highest cancer mortality rates were observed for the age group 35–39 years (19.9 [19.4, 20.5] during 1980–2019), and for malignant neoplasms of the brain and CNS (1.2 [1.2, 1.3]) and the breast (1.1 [1.0, 1.2]), and for leukemias (1.1 [1.0, 1.2]).

**Table 3:** Observed number of cancer deaths (n), age-standardized mortality (per 100′000 person years) and average annual percent change (AAPC) of cancer in AYAs in Switzerland for different time periods between 1980 and 2019 by language region, sex, age at death and cancer death category

Characteristics		Observed number of cancer deaths (n)					Age-standardized mortality rate per 100'000 person years					AAPC [95% CI]
		Total^1^ (%)	1980- 1989	1990- 1999	2000- 2009	2010- 2019	Total^1^	1980- 1989	1990- 1999	2000- 2009	2010- 2019	1980–2019
All cancer deaths		9′865 (100)	3′341	2′722	2′091	1′711	9.2	13.3	10.0	7.7	6.2	−2.5 [−2.7, −2.3]
Language region												
German		7′123 (72)	2′438	1′983	1′514	1′188	9.4	13.4	10.3	8.0	6.2	−2.5 [−2.7, −2.3]
French/Italian		2′742 (28)	903	739	577	523	8.8	12.8	9.5	7.2	6.3	−2.5 [−2.8, −2.2]
Sex												
Female		4′910 (50)	1′593	1′370	1′079	868	9.2	12.8	10.1	7.9	6.3	−2.3 [−2.6, −2.1]
Male		4′955 (50)	1′748	1′352	1′012	843	9.3	13.7	10.0	7.6	6.1	−2.7 [−2.9, −2.4]
Age at death (years)												
15–19		706 (7.2)	280	158	129	139	4.0	5.7	3.9	3.0	3.2	−1.4 [−2.8, −0.1]
20–24		936 (9.5)	357	240	202	137	4.9	7.0	5.2	4.6	2.8	−2.5 [−3.1, −1.9]
25–29		1′370 (14)	472	393	262	243	6.6	9.5	7.1	5.5	4.4	−2.6 [−3.0, −2.2]
30–34		2′360 (24)	780	683	483	414	10.7	15.5	11.6	8.9	7.2	−2.5 [−2.8, −2.1]
35–39		4′493 (46)	1′452	1′248	1′015	778	19.9	28.6	22.4	16.7	13.5	−2.6 [−2.8, −2.3]
Cancer death category												
Malignant neoplasms												
Lip, oral cavity, and pharynx		156 (1.6)	51	45	37	23	0.1	0.2	0.2	0.1	0.1	−2.0 [−3.3, −0.6]
Oesophagus		69 (0.7)	20	21	13	15	0.1	0.1	0.1	0.0	0.1	−1.7 [−3.0, −0.4]
Stomach		415 (4.2)	112	113	104	86	0.4	0.4	0.4	0.4	0.3	−1.2 [−2.2, −0.3]
Colon		395 (4.0)	109	92	100	94	0.4	0.4	0.3	0.4	0.3	−0.9 [−1.8, −0.1]
Rectosigmoid junction and rectum		150 (1.5)	36	41	33	40	0.1	0.1	0.1	0.1	0.1	−0.6 [−1.7, 0.6]
Liver and intrahepatic bile ducts		195 (2.0)	41	55	52	47	0.2	0.2	0.2	0.2	0.2	−0.4 [−1.4, 0.5]
Biliary tract		29 (0.3)	11	8	6	4	0.0	0.0	0.0	0.0	0.0	−2.4 [−4.6, −0.1]
Pancreas		217 (2.2)	58	61	46	52	0.2	0.2	0.2	0.2	0.2	−0.4 [−1.7, 0.9]
Larynx		14 (0.1)	6	2	5	1	0.0	0.0	0.0	0.0	0.0	−2.0 [−6.0, 2.2]
Trachea, bronchus, and lung		613 (6.2)	209	178	122	104	0.6	0.8	0.6	0.4	0.4	−5.0 [−10.5, 0.8]
Pleura mesothelioma		37 (0.4)	24	12	0	1	0.0	0.1	0.0	0.0	0.0	−5.6 [−9.6, −1.3]
Melanoma of skin		580 (5.9)	182	174	132	92	0.5	0.7	0.6	0.5	0.3	−2.5 [−3.4, −1.7]
Breast		1′207 (12)	387	362	264	194	1.1	1.5	1.3	0.9	0.7	−2.7 [−3.2, −2.3]
Cervix uteri		289 (2.9)	122	85	37	45	0.3	0.5	0.3	0.1	0.2	−3.6 [−4.5, −2.6]
Corpus uteri		45 (0.5)	15	6	12	12	0.0	0.1	0.0	0.0	0.0	−2.1 [−3.8, −0.3]
Ovary		193 (2.0)	64	53	45	31	0.2	0.3	0.2	0.2	0.1	−1.5 [−2.8, −0.2]
Prostate		8 (0.0)	4	2	1	1	0.0	0.0	0.0	0.0	0.0	−2.7 [−6.4, 1.0]
Testis		371 (3.8)	178	108	46	39	0.3	0.7	0.4	0.2	0.1	−5.4 [−6.3, −4.5]
Kidney		106 (1.1)	35	26	24	21	0.1	0.1	0.1	0.1	0.1	−2.1 [−3.6, −0.6]
Bladder		47 (0.5)	15	11	8	13	0.0	0.1	0.0	0.0	0.1	−1.1 [−2.6, 0.3]
Brain and CNS		1′297 (13)	382	335	300	280	1.2	1.5	1.3	1.1	1.0	−1.3 [−1.8, −0.8]
Thyroid		21 (0.2)	5	9	4	3	0.0	0.0	0.0	0.0	0.0	−0.8 [−2.6, 1.1]
Hodgkin lymphoma		369 (3.7)	213	93	44	19	0.4	0.9	0.4	0.2	0.1	−6.8 [−8.0, −5.7]
Non-Hodgkin lymphoma		540 (5.5)	195	180	96	69	0.5	0.8	0.7	0.4	0.3	−3.4 [−4.3, −2.6]
Multiple myeloma		36 (0.4)	11	5	11	9	0.0	0.0	0.0	0.0	0.0	0.4 [−1.3, 2.3]
Leukemia		1′108 (11)	421	308	229	150	1.1	1.7	1.2	0.9	0.6	−3.7 [−2.6, −11.4]
Other and unspecified malignant neoplasms		1′251 (13)	383	313	296	259	1.2	1.5	1.2	1.2	1.0	−1.3 [−1.7, −0.8]
Benign or uncertain neoplasms of brain and CNS		107 (1.1)	52	24	24	7	0.1	0.2	0.1	0.1	0.0	−4.8 [−6.2, −3.4]

*AAPC* average annual percent change; *CNS* central nervous system

^1^ The total corresponds to the whole of the study period, 1980–2019

### Trends

**Fig. 2:**
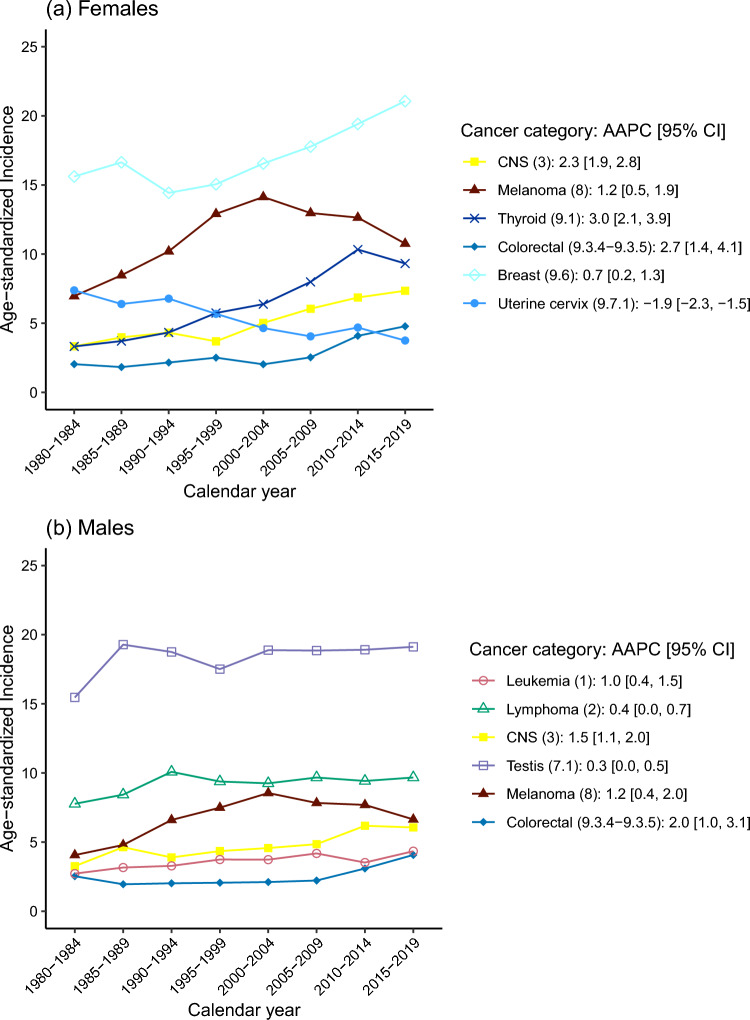
Trends in age-standardized incidence per 100′000 person years for the most frequent AYA cancers (cancer category number according to Barr et al.[24]) in females (**a**) and males (**b**) in Switzerland between 1980 and 2019

Focusing on the most frequent cancer categories by sex (breast, testis, melanoma, CNS, colorectal, thyroid, leukemia, lymphoma, and uterine cervix), we observed the most marked average annual increases in incidence for thyroid carcinoma in females (AAPC 3.0% [2.1, 3.9]), although this seems to have leveled off in the most recent years, and for colorectal carcinoma in both sexes (females 2.7% [1.4, 4.1]; males 2.0% [1.0, 3.1]) (Fig. 2). Particularly steep increases for colorectal carcinoma occurred in recent years (2010–2019) and at higher ages (30–39 years) (Figs. 2 and S1). Notable increases were also observed in the incidence of CNS neoplasms in both sexes (AAPC females: 2.3% [1.9, 2.8], males: 1.5% [1.1, 2.0]). In females, we estimated an average annual increase of 0.7% [0.2, 1.3] in the incidence of breast carcinoma (initially decreasing until 1993 and increasing by 2% per year thereafter; Figure S2), and an average annual decline of -1.9% [-2.3, -1.5] for uterine cervix carcinoma. In males, leukemia incidence rose by 1% [0.4, 1.5] on average per year whereas incidence of testicular cancer stayed relatively stable (AAPC 0.3% [0.0, 0.5]). We observed a trend reversal for melanoma in both sexes (increasing during the early phase of the study period, peaking in 2002, and declining thereafter; Figure S3) (AAPC males: 1.2% [0.4, 2.0], females: 1.2% [0.5, 1.9]). The calendar year of this reversal appeared to increase with age (Figure S4). In males, we observed a trend reversal for non-Hodgkin lymphomas (increasing by 7% per year until 1991 and declining by −1.2% annually thereafter) but not for Hodgkin lymphomas (Fig. S5). An initially increasing trend that declined steeply after 1995 was observed for Kaposi sarcomas in males (Table S5 and Figure S6).

**Fig. 3:**
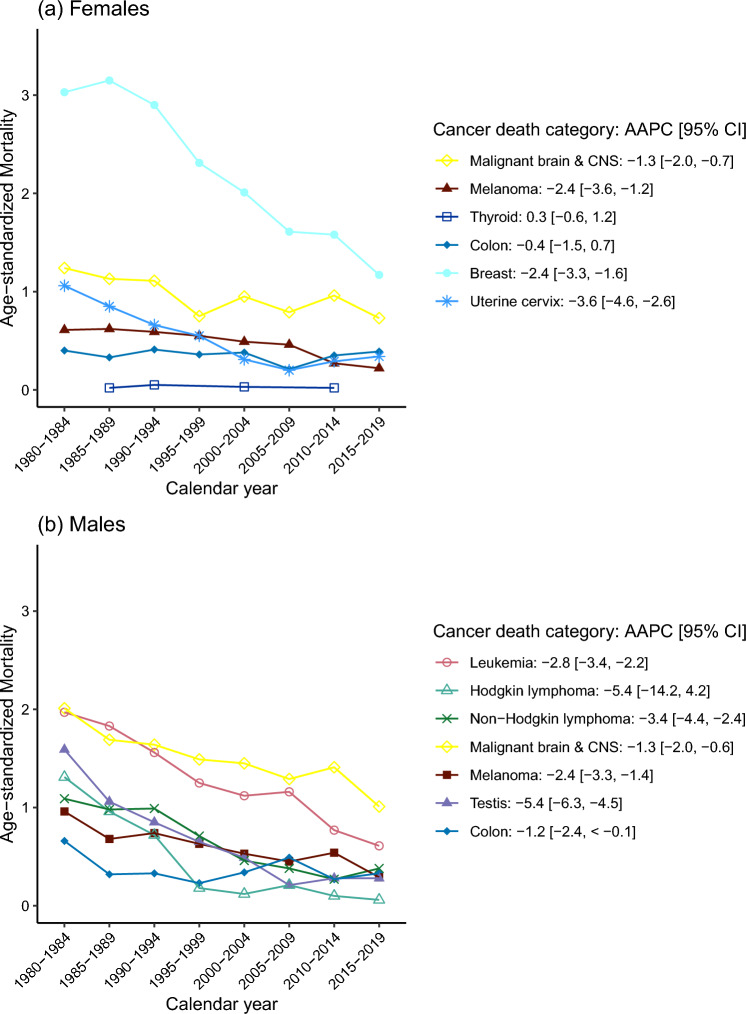
Trends in age-standardized mortality per 100′000 person years for the most frequent AYA cancers in females (**a**) and males (**b**) in Switzerland between 1980 and 2019

Overall, cancer mortality rates fell by 2.5% ([−2.7, −2.3]) on average per year during 1980–2019 (Table 3). The most marked average declines were observed for tumors of uterine cervix in females (AAPC—3.6% [-4.6, -2.6]) and, in males, for testicular cancer (−5.4% [−6.3, −4.5]) and non-Hodgkin lymphoma (−3.4 [−4.4, −2.4]) (Fig. 3). We observed no or only a weak decline for malignant neoplasms of the thyroid in females (AAPC 0.3% [-0.6, 1.2]) and of the colon in both sexes (AAPC males: -1.2% [−2.4, 0.0], females: -0.4% [−1.5, 0.7]). We observed a halt in the decline of overall cancer mortality among 15- to 19-year olds after 2006 (Figs. S8) and, among males, for Hodgkin lymphoma after 1996 (Fig. S9).

Sensitivity analyses restricting the study population to seven cantons with continuous registration during the study period showed closely similar incidence and mortality trends as our main analyses (Suppl. Figures S7 and S10).

## Discussion

This first nationwide study examining incidence, mortality, and cumulative risk of AYA cancers in Switzerland revealed an overall increase in cancer in both sexes and steadily decreasing mortality from all cancers during 1980–2019. The most common cancers included female breast carcinoma, testicular cancer, melanomas, colorectal carcinomas, lymphomas, and CNS neoplasms. The incidence of colorectal carcinomas and CNS neoplasms increased in both sexes over the study period, whereas melanoma incidence declined after peaking in the early to mid 2000s. Notably, we observed strong upward trends for thyroid and breast carcinoma incidence in females, and a declining trend for carcinoma of uterine cervix. Based on incidence and mortality data from 2015–2019 (including childhood cancers), the estimated risk of developing a cancer before the age of 40 was 2.0%.

The trends of AYA cancer in Switzerland align with patterns of increasing incidence rates [11–13] and decreasing mortality rates [13, 16] reported for neighboring countries. In Italy, Germany, and France overall age-standardized incidence rates are similar to those we found in Switzerland [13]. The increasing incidence of thyroid and breast carcinomas in women and of colorectal carcinomas and CNS neoplasms in both sexes that we observed in Switzerland mirror trends observed in other European countries [13] and globally for colorectal and thyroid carcinomas [35]. A trend reversal as we observed for melanoma incidence was similarly reported for AYAs in Australia and America [11].

The favorable trends for cancer mortality, already observed in a Swiss study from 1991, can largely be attributed to advances in therapy [18]. However, other factors are also likely to have contributed to these observed declines. Earlier diagnosis through screening and increased awareness has led to the detection of less advanced diseases and, in some cases, prevented cancers entirely [36]. Improved access to multidisciplinary care and the implementation of preventive vaccination programs may also have contributed to these favorable trends [37]. Previous research showed that although high Socio-demographic Index (SDI) countries such as Switzerland had the highest age-standardized incidence rates, they also had the lowest age-standardized mortality rates compared to lower SDI countries [38]. Similarities between Switzerland and other high-income countries may reflect comparable lifestyle patterns, cancer prevention and management strategies, and healthcare infrastructure. We found similar cumulative risks for AYA cancer as reported by an earlier Swiss study [39].

The observed increases in cancer incidence may have multiple causes, including increased exposure to cancer risk factors, changes in lifestyle and screening practices, overdiagnosis, and the introduction of cancer preventive strategies. Improvements in cancer registration in Switzerland appear to have primarily led to better case-level data quality rather than to the detection of more cancers [22]. So, we believe that they do not explain the observed increases in cancer incidence.

The driving factors for rising trends in the incidence of breast carcinoma in AYAs remain unclear. Screening is an unlikely explanation as programs target women at higher age; published guidelines recommend routine screening for breast cancer in average-risk women above the age of 50 years [40, 41]. Nevertheless, a recently conducted survey in Switzerland showed that women aged 30–49 engage relatively often in opportunistic mammography screening [42]. Potential risk factors in young women include family history, higher age at first childbearing, low body mass index, and oral contraceptive use [43]. Declining trends in incidence for uterine cervical carcinoma, primarily caused by sexually transmitted human papillomavirus (HPV), are more likely related to screening and behavioral risk factors (e.g., sexual behavior) than to preventive HPV vaccination, introduced in Switzerland in 2008 [44, 45]. Colorectal cancer incidence has been associated with genetic mutations and prior diseases (e.g. Lynch syndrome caused by germline mutations), but the rising trend could potentially be linked to lifestyle and dietary factors (e.g. red meat consumption or sedentary lifestyle) [46]. In particular, a higher risk of colorectal cancer was observed in younger adults who were overweight or obese [47]. Interestingly, in some screening cohort overweight or obese individuals presented with less advanced stages at diagnosis [48], suggesting that the putative contribution of obesity on rising trends may be partially mediated by alterations in stage distribution. Testicular cancer is the most common cancer in male AYAs and the persistently high rates in our study confirm results from other European countries [13] and international findings [49].

Despite the relatively high incidence rates of melanoma, which were almost double in females compared to males, a decline has been observed in AYAs aged below 35 years since 2002. Melanoma in AYAs appears to be associated with increased ultraviolet light exposure at a young age [50]. The declining incidence may be due to increased awareness through organized prevention programs [51, 52] or other successful interventions to reduce indoor tanning and increase sun-protective behaviors [13, 53], particularly in younger populations. The earlier trend reversal observed in our study among younger AYAs may reflect a comparably greater impact of sun-protection behaviors in childhood and adolescence as well as effective prevention programs specifically targeted to this group.

The globally increasing trend in thyroid carcinoma among AYAs, observed also by our study, has been attributed to overdiagnosis, suggesting that some of these cancers may not have affected the persons’ well-being during their lifetime if left undetected [54]. A previous Swiss study also highlighted the overdiagnosis and overtreatment in Switzerland between 1998 and 2012 [55]. Moreover, research has shown that most untreated, non-metastasized, localized papillary thyroid cancers are non-aggressive, particularly in younger patients with small tumors [56]. This underscores the need to interpret cancer trends cautiously in the context of changing diagnostic practices and to differentiate between aggressive and non-aggressive cancers.

In our study, the incidence of Kaposi sarcoma peaked in male AYAs during the 1980s and early 1990s (Table S5 and Figure S6) in parallel with the human immunodeficiency virus (HIV) and acquired immune deficiency syndrome (AIDS) epidemic [57]. After the epidemic, international trends of incidence among AYAs stabilized at a lower but still significant rate and gradually declined due to improved antiretroviral treatment and HIV control measures [12]. Furthermore, the introduction and broad uptake of global expansion of highly active antiretroviral therapy (HAART) have been associated with substantial reductions in Kaposi sarcoma incidence and improvements in survival among people living with HIV [58]. More recent innovations, such as pre-exposure prophylaxis (PrEP), include both daily oral and long-acting injectable antiretroviral formulations (e.g., cabotegravir) that have demonstrated efficacy in preventing HIV acquisition and thus may thus help stem the epidemic [59–61].

The observed increase in incidence of CNS tumors may in part be explained by the rise in the use of medical imaging resulting in incidental findings [62].

Our results highlight that the spectrum of cancers in AYAs differs significantly from other age groups [1–3]. In AYAs, carcinomas account for around one third of all cancers, followed by gonadal tumors, lymphomas, and melanomas. In contrast, in children leukemias and embryonal tumours predominate, while in older adults carcinomas account for over 80% of cases [1–3]. This underscores the need to differentiate AYA cancers from those in children and older adults. While childhood cancers are often related to congenital factors, and adult cancers to environmental and lifestyle factors, AYA cancers may be due to a combination of these [63]. Cancer diagnoses among AYAs may be delayed due to a lack of knowledge about cancer and its symptoms [64], or simply because of the perception that cancer is uncommon at that age. Thus, raising awareness about cancer in this age group and promoting a healthy lifestyle is crucial [35].

Moreover, regional variation in cancer incidence within Switzerland should be considered. Lifestyle and socio-cultural differences may partly explain the higher incidence in the Italian- and French-speaking regions. For example, a Swiss study found that men from the Italian-speaking region were almost three times more likely to be physically inactive than their German-speaking counterparts [65]. Similarly, attitudes towards healthcare, participation and availability in screening programs could influence early detection and thus reported incidence rates, as evidenced by a Swiss study that found markedly higher mammography attendance rates in the French-speaking region (77.8%) than in the German-speaking region (34.9%) [66].

This is the first comprehensive epidemiological investigation of cancer in AYAs in Switzerland covering the entire country and all cancer types. Our study made use of all available data from existing cancer registries and covered a period of four decades (1980–2019). In addition, the study is based on a cancer classification specifically developed for the AYA age period [24].

Our study was limited by the incomplete coverage of cancer registration, particularly during the early phase of our study period. In Switzerland, cancer registration became mandatory only in 2020. Incidence estimates derived from registry data could be biased if the underlying cancer rates in areas not covered by registration differ from those in areas covered. However, sensitivity analyses restricting to seven cantons with continuous cancer registration throughout our study period showed closely similar incidence and mortality estimates as the main analyses (Suppl. Figs. S7 and S10). This suggests that incidence rates in cantons with incomplete registration between 1980 and 2019 were well represented by cantons with long-standing cancer registration during the same period. The representativeness of our study may have been affected by differences in the completeness of case ascertainment between different cancer registries and/or between different diagnostic groups [22]. Comparisons of incidence with mortality rates were limited by the different classification and coding systems used (i.e. ICD-O versus ICD-10, and carcinomas versus malignant neoplasms). However, for many of the most frequent cancer types, including testicular, breast, thyroid, colorectal and cervical cancer, Hodgkin and non-Hodgkin lymphoma, and melanoma, 94%-100% of incident cases were mapped to the same categories used in the mortality analyses. Somewhat lower agreement was found for CNS tumors (87%) and for Leukemia (77%), which was mostly attributed to inconsistencies in coding between the different systems.

## Conclusion

This nationwide study on AYA cancer in Switzerland confirmed the composition of cancer types for this age group and found comparable trends in incidence and mortality as previous studies in other high-income countries have reported. Notably, we observed increasing trends in the overall incidence of AYA cancer over time, and specifically for thyroid and colorectal carcinomas, CNS neoplasms, melanoma, and lymphomas. Further research is needed to understand the drivers of these observed trends. Mortality rates have generally decreased, reflecting improvements in cancer management. Our study underscores the importance of tailored age-specific interventions for AYA cancers to improve the care and meet the needs of AYA cancer survivors [67], highlighting the need for continued surveillance and prevention strategies.

## Supplementary Information

Below is the link to the electronic supplementary material.

Supplementary file1 (DOCX 1577 KB)

Original Supplementary Material with reformatting revisions

Original Supplementary Material with reformatting revisions in Track changes format

## Data Availability

The datasets used and/or analysed during the current study can be requested from the ChCR and from the NKRS directly on reasonable request, as the authors of this paper are not allowed to share the data themselves.

## Abbreviations

AAPC: Average annual percent change
AIDS: Acquired immune deficiency syndrome
AYAs: Adolescents and young adults
CCR: Cantonal cancer registries
ChCR: Childhood Cancer Registry
CIs: Confidence intervals
CNS: Central nervous system
FSO: Swiss Federal Statistical Office
HAART: Highly active antiretroviral therapy
HIV: Human immunodeficiency virus
HPV: Human papillomavirus
ICD: International Statistical Classification of Diseases and Related Health Problems
ICD-O: International Classification of Diseases for Oncology
NCI: National Cancer Institute
NIH: National Institutes of Health
NACR: National Agency for Cancer Registration
PrEP: Pre-exposure prophylaxis
SDI: Socio-demographic Index
