# Supplementary material for: Incidence, mortality, and cumulative risk of cancer in adolescents and young adults in Switzerland
Source: Eur J Epidemiol. 2026 Feb 21;41(4):459–77. doi: 10.1007/s10654-026-01363-9 (PMC13331890; doi:10.1007/s10654-026-01363-9)
Supplement: Supplementary file 1 — Supplementary file1 (DOCX 1577 KB) [file 10654_2026_1363_MOESM1_ESM.docx]

Appendix to the manuscript

**Incidence, mortality, and cumulative risk of cancer**

**in adolescents and young adults (AYAs) in Switzerland**

Céline Bolliger, Eleftheria Michalopoulou, Christian Kreis, Matthias Lorez, Christina Schindera, Benjamin Kasenda, Claudia E. Kuehni, Daniela Dyntar, Katharina Roser*, Ben D. Spycher*

*Shared last authorship

Corresponding author: Ben D. Spycher

**Supplementary Material**

| **Table S1** | Start Years of Data Registration in Swiss Cantonal Cancer Registries |
| --- | --- |
| **Table S2** | Age-Standardized Cancer Incidence in AYAs in Switzerland by Region, Age, and Cancer Category (1980-2019) |
| **Table S3** | Age-Standardized Cancer Mortality in AYAs in Switzerland by Region, Age, and Cancer Category (1980-2019) |
| **Table S4** | Estimated Primary Cancer Numbers and Incidence in Female AYAs in Switzerland by Region, Age, and Cancer Category (1980-2019) |
| **Table S5** | Estimated Primary Cancer Numbers and Incidence in Male AYAs in Switzerland by Region, Age, and Cancer Category (1980-2019) |

| **Figure S1** | Trends in Age-Specific Incidence for Colorectal Carcinoma in Switzerland (1980-2019) |
| --- | --- |
| **Figure S2** | Trends in Age-Standardized Incidence for Female Breast Carcinoma in Switzerland (1980-2019) |
| **Figure S3** | Trends in Age-Standardized Incidence for Malignant Melanoma in Females and Males in Switzerland (1980-2019) |
| **Figure S4** | Trends in Age-Specific Incidence for Malignant Melanoma in Switzerland (1980-2019) |
| **Figure S5** | Trends in Age-Standardized Incidence for Non-Hodgkin and Hodgkin Lymphoma in Males in Switzerland (1980-2019) |
| **Figure S6** | Trends in Age-Standardized Incidence for Kaposi Sarcoma in Males in Switzerland (1980-2019) |
| **Figure S7** | Figure S7. Sensitivity analyses of incidence trends restricting to seven cantons with continuous registration during the study period (old registries). |
| **Figure S8** | Trends in Age-Specific Mortality in Switzerland (1980-2019) |
| **Figure S9** | Trends in Age-Standardized Mortality for Hodgkin Lymphoma in Males in Switzerland (1980-2019) |
| **Figure S10** | Figure S7. Sensitivity analyses of mortality trends restricting to seven cantons with continuous registration during the study period (old registries). |

**Table S1**. Start years of data registration in Swiss cantonal cancer registries.

| **Canton and Cancer Registry** | **Start of data registration** |
| --- | --- |
| **Geneva**  Registre genevois des tumeurs | 1970 |
| **Neuchâtel**  Registre neuchâtelois et jurassien des tumeurs | 1974 |
| **Vaud**  Registre vaudois des tumeurs | 1974 |
| **St. Gallen**  Krebsregister Ostschweiz | 1980 |
| **Zurich**  Krebsregister der Kantone Zürich, Zug, Schaffhausen und Schwyz | 1980 |
| **Appenzell Innerrhoden**  Krebsregister Ostschweiz | 1980 |
| **Appenzell Ausserrhoden**  Krebsregister Ostschweiz | 1980 |
| **Basel-Landschaft**  Krebsregister beider Basel | 1981 |
| **Basel-Stadt**  Krebsregister beider Basel | 1981 |
| **Valais**  Walliser Krebsregister | 1989 |
| **Graubünden**  Krebsregister der Kantone Graubünden und Glarus | 1989 |
| **Glarus**  Krebsregister der Kantone Graubünden und Glarus | 1992 |
| **Ticino**  Registro cantonale dei tumori | 1996 |
| **Jura**  Registre neuchâtelois et jurassien des tumeurs | 2005 |
| **Fribourg**  Krebsregister Freiburg | 2006 |
| **Lucerne**  Zentralschweizer Krebsregister | 2010 |
| **Zug**  Krebsregister der Kantone Zürich, Zug, Schaffhausen und Schwyz | 2011 |
| **Nidwalden**  Zentralschweizer Krebsregister | 2011 |
| **Uri**  Zentralschweizer Krebsregister | 2011 |
| **Obwalden**  Zentralschweizer Krebsregister | 2011 |
| **Thurgau**  Krebsregister Ostschweiz | 2012 |
| **Aargau**  Krebsregister Aargau | 2013 |
| **Bern**  Krebsregister Bern Solothurn | 2014 |
| **Solothurn**  Krebsregister Bern Solothurn | 2019 |
| **Schwyz**  Krebsregister der Kantone Zürich, Zug, Schaffhausen und Schwyz | 2020 |
| **Schaffhausen**  Krebsregister der Kantone Zürich, Zug, Schaffhausen und Schwyz | 2020 |

Note: Data from the Federal Statistical Office [1]

**Table S2**. Age-standardized incidence (per 100’000 person years and according to the 2013 European standard population) of cancer in AYAs in Switzerland for different time periods between 1980 and 2019 by language region, age at diagnosis, and cancer category.

| **Population characteristics** | **Age-standardised incidence** | | | | |
| --- | --- | --- | --- | --- | --- |
|  | **Total^1^** | **1980-1989** | **1990-1999** | **2000-2009** | **2010-2019** |
| **All cancers** | 68.9 | 59.3 | 66.4 | 69.2 | 75.3 |
| **Language region** |  |  |  |  |  |
| German | 68.5 | 59.3 | 66.8 | 67.8 | 74.6 |
| French/Italian | 69.8 | 59.3 | 65.6 | 72.3 | 76.8 |
| **Sex** |  |  |  |  |  |
| Female | 74.9 | 62.5 | 69.3 | 75.6 | 84.4 |
| Male | 62.9 | 56.2 | 63.7 | 62.8 | 66.2 |
| **Cancer category^2,3^** |  |  |  |  |  |
| 1. Leukemias and related disorders | 3.2 | 2.6 | 2.9 | 3.5 | 3.6 |
| 1.1 Acute lymphoblastic leukemia | 0.6 | 0.6 | 0.6 | 0.7 | 0.7 |
| 1.2 Acute myeloid leukemia | 1.0 | 1.0 | 1.1 | 1.1 | 1.0 |
| 1.3 Chronic myeloid leukemia | 0.6 | 0.5 | 0.6 | 0.6 | 0.5 |
| 1.6 Essential thrombocythemia | 0.3 | 0.1 | 0.1 | 0.2 | 0.5 |
| 2. Lymphomas | 8.1 | 6.8 | 8.4 | 8.4 | 8.5 |
| 2.1 Non-Hodgkin lymphomas | 3.3 | 2.5 | 3.9 | 3.5 | 3.3 |
| 2.1.2 Burkitt | 0.3 | 0.2 | 0.3 | 0.3 | 0.2 |
| 2.1.3 Diffuse large B-cell (DLBCL) | 1.3 | 0.9 | 1.8 | 1.4 | 1.1 |
| 2.1.5 Anaplastic T-cell and null-cell excluding NK/T-cell | 0.5 | 0.3 | 0.4 | 0.5 | 0.6 |
| 2.1.6 Follicular | 0.4 | 0.3 | 0.4 | 0.5 | 0.5 |
| 2.2 Hodgkin lymphoma | 4.2 | 3.4 | 3.8 | 4.5 | 4.7 |
| 2.2.2 Hodgkin classic, other | 4.0 | 3.4 | 3.8 | 4.2 | 4.4 |
| 3. CNS and other intracranial and intraspinal neoplasms | 5.3 | 3.9 | 4.2 | 5.3 | 6.8 |
| 3.1 Astroglial and related neoplasms | 2.8 | 2.4 | 2.5 | 2.9 | 3.1 |
| 3.1.1 Oligodendriogliomas | 0.6 | 0.4 | 0.4 | 0.7 | 0.7 |
| 3.1.2 Glioblastomas/gliofibromas | 0.5 | 0.3 | 0.4 | 0.5 | 0.5 |
| 3.1.3 Ependymomas | 0.4 | 0.3 | 0.2 | 0.4 | 0.6 |
| 3.4 Neuronal and mixed neuronal-glial neoplasms | 0.3 | 0.1 | 0.1 | 0.3 | 0.4 |
| 3.4.1 Neuronal and mixed neuronal-glial, benign/borderline | 0.3 | 0.1 | 0.1 | 0.3 | 0.4 |
| 3.5 Meningiomas | 1.1 | 0.5 | 0.7 | 1.0 | 1.6 |
| 3.5.1 Meningioma, benign/borderline | 1.0 | 0.5 | 0.7 | 0.9 | 1.6 |
| 3.8 Pituitary neoplasms | 0.7 | 0.4 | 0.4 | 0.6 | 1.0 |
| 3.8.1 Pituitary, benign/borderline | 0.6 | 0.3 | 0.3 | 0.6 | 1.0 |
| 4. Sarcomas | 2.9 | 2.6 | 3.0 | 2.8 | 3.0 |
| 4.1 Osteosarcoma | 0.4 | 0.3 | 0.4 | 0.4 | 0.3 |
| 4.2 Chondrosarcoma | 0.3 | 0.2 | 0.3 | 0.3 | 0.3 |
| 4.3 Ewing family of tumors | 0.3 | 0.3 | 0.3 | 0.2 | 0.4 |
| 4.4 Fibromatous neoplasms | 0.6 | 0.6 | 0.8 | 0.5 | 0.5 |
| 4.5 Liposarcoma | 0.3 | 0.3 | 0.2 | 0.3 | 0.3 |
| 5. Blood and lymphatic vessel tumor | 1.1 | 1.3 | 2.1 | 0.8 | 0.5 |
| 5.2 Malignant blood and lymphatic vessel tumors, all sites | 0.9 | 1.1 | 2.0 | 0.5 | 0.3 |
| 5.2.1 Kaposi sarcoma | 0.8 | 1.0 | 1.9 | 0.4 | 0.2 |
| 6. Nerve sheath tumors | 0.6 | 0.3 | 0.5 | 0.5 | 0.9 |
| 6.1 Benign, CNS | 0.5 | 0.2 | 0.4 | 0.4 | 0.7 |
| 6.1.1 Neurilemmoma | 0.5 | 0.2 | 0.4 | 0.4 | 0.7 |
| 7. Gonadal and related tumors | 11.1 | 10.7 | 11.1 | 11.0 | 11.3 |
| 7.1 Testis | 9.6 | 8.9 | 9.4 | 9.7 | 9.9 |
| 7.1.1 Germ cell and trophoblastic | 9.6 | 8.9 | 9.4 | 9.7 | 9.9 |
| 7.2 Ovary | 1.1 | 1.3 | 1.3 | 0.9 | 1.0 |
| 7.2.1 Germ cell and trophoblastic | 0.3 | 0.3 | 0.3 | 0.2 | 0.3 |
| 7.2.2 Non-germ cell | 0.8 | 1.0 | 1.0 | 0.7 | 0.7 |
| 7.4 Germ cell and trophoblastic excluding CNS, ovary, testis | 0.3 | 0.4 | 0.3 | 0.3 | 0.3 |
| 8. Melanoma, malignant | 9.5 | 6.4 | 9.6 | 11.3 | 9.8 |
| 8.1 Superficial spreading/low cumulative sun damage melanoma | 5.7 | 3.2 | 5.7 | 6.8 | 6.1 |
| 8.2 Nodular melanoma | 0.9 | 1.2 | 1.1 | 0.9 | 0.5 |
| 9. Carcinomas | 26.6 | 24.1 | 24.1 | 25.2 | 30.5 |
| 9.1 Thyroid carcinoma | 4.7 | 2.4 | 3.5 | 4.9 | 6.6 |
| 9.1.3 Papillary | 2.8 | 1.0 | 1.5 | 3.1 | 4.4 |
| 9.1.4 Follicular | 0.6 | 0.3 | 0.6 | 0.7 | 0.7 |
| 9.1.5 Papillary with follicular variant | 1.0 | 0.8 | 1.1 | 0.9 | 1.3 |
| 9.2 Other carcinoma of head and neck | 1.4 | 1.5 | 1.5 | 1.3 | 1.2 |
| 9.2.2 Oral cavity, lip, and pharynx | 0.7 | 0.9 | 0.7 | 0.7 | 0.6 |
| 9.2.3 Salivary gland | 0.2 | 0.1 | 0.3 | 0.2 | 0.3 |
| 9.3 Carcinoma of gastrointestinal tract | 5.0 | 4.2 | 4.5 | 4.2 | 6.3 |
| 9.3.2 Carcinoma of stomach | 0.9 | 1.0 | 1.0 | 0.8 | 0.8 |
| 9.3.4 Carcinoma of colon | 2.1 | 1.4 | 1.5 | 1.6 | 3.1 |
| 9.3.5 Carcinoma of rectum | 0.9 | 0.8 | 0.9 | 0.8 | 1.1 |
| 9.3.7 Carcinoma of liver and intrahepatic bile ducts (IBD) | 0.3 | 0.2 | 0.3 | 0.3 | 0.3 |
| 9.3.9 Carcinoma of pancreas | 0.4 | 0.3 | 0.4 | 0.3 | 0.4 |
| 9.4 Carcinoma of lung, bronchus, and trachea | 1.2 | 1.6 | 1.4 | 1.0 | 1.1 |
| 9.4.2 Non-small cell carcinoma | 1.1 | 1.3 | 1.2 | 0.9 | 1.1 |
| 9.6 Carcinoma of breast | 9.5 | 8.8 | 8.0 | 9.4 | 11.0 |
| 9.6.1 Breast, infiltrating duct | 8.0 | 5.9 | 6.5 | 8.1 | 9.8 |
| 9.6.2 Breast, adenocarcinoma | 0.5 | 1.4 | 0.2 | 0.3 | 0.3 |
| 9.6.3 Breast, lobular | 0.4 | 0.5 | 0.5 | 0.5 | 0.3 |
| 9.6.5 Breast, medullary | 0.3 | 0.4 | 0.3 | 0.3 | 0.2 |
| 9.7 Carcinoma of genital sites excluding ovary and testis | 3.4 | 4.2 | 3.9 | 3.1 | 2.7 |
| 9.7.1 Carcinoma of uterine cervix | 2.7 | 3.7 | 3.3 | 2.4 | 2.2 |
| 9.7.2 Corpus uteri | 0.4 | 0.3 | 0.4 | 0.5 | 0.4 |
| 9.8 Carcinoma of urinary tract | 1.0 | 1.0 | 0.9 | 0.9 | 1.1 |
| 9.8.1 Carcinoma of kidney | 0.7 | 0.5 | 0.6 | 0.7 | 0.9 |
| 9.8.2 Carcinoma of bladder | 0.3 | 0.5 | 0.3 | 0.2 | 0.2 |
| 10. Miscellaneous specified neoplasms | 0.2 | 0.3 | 0.3 | 0.3 | 0.2 |
| 11. Unspecified malignant neoplasms except CNS | 0.3 | 0.4 | 0.3 | 0.2 | 0.2 |

Abbreviations: **CNS**, central nervous system

^1^ The total corresponds to the whole of the study period, 1980-2019

^2^ Cancer categories according to Barr et al.[2]

^3^ Cancer categories reported only if included in Table 1

**Table S3**. Age-standardized mortality (per 100’000 person years and according to the 2013 European standard population) of cancer in AYAs in Switzerland for different time periods between 1980 and 2019 by language region, age at diagnosis, and cancer category.

|  | **Population characteristics** | **Age-standardised mortality** | | | | |
| --- | --- | --- | --- | --- | --- | --- |
|  |  | **Total^1^** | **1980-1989** | **1990-1999** | **2000-2009** | **2010-2019** |
| **All cancer deaths** | | 9.7 | 13.9 | 10.6 | 8.2 | 6.5 |
| **Language region** | |  |  |  |  |  |
| German | | 9.9 | 14.1 | 10.8 | 8.4 | 6.5 |
| French/Italian | | 9.3 | 13.4 | 10.0 | 7.6 | 6.6 |
| **Sex** | |  |  |  |  |  |
| Female | | 9.7 | 13.5 | 10.8 | 8.4 | 6.7 |
| Male | | 9.6 | 14.3 | 10.4 | 7.9 | 6.4 |
| **Cancer death category** | |  |  |  |  |  |
| **Malignant neoplasm** | Lip, oral cavity, and pharynx | 0.2 | 0.2 | 0.2 | 0.1 | 0.1 |
|  | Oesophagus | 0.1 | 0.1 | 0.1 | 0.1 | 0.1 |
|  | Stomach | 0.4 | 0.5 | 0.4 | 0.4 | 0.3 |
|  | Colon | 0.4 | 0.5 | 0.4 | 0.4 | 0.4 |
|  | Rectosigmoid junction and rectum | 0.2 | 0.2 | 0.2 | 0.1 | 0.2 |
|  | Liver and intrahepatic bile ducts | 0.2 | 0.2 | 0.2 | 0.2 | 0.2 |
|  | Biliary tract | 0.0 | 0.1 | 0.0 | 0.0 | 0.0 |
|  | Pancreas | 0.2 | 0.3 | 0.2 | 0.2 | 0.2 |
|  | Larynx | 0.0 | 0.0 | 0.0 | 0.0 | 0.0 |
|  | Tachea, bronchus, and lung | 0.6 | 0.9 | 0.7 | 0.5 | 0.4 |
|  | Pleuramesotheliom | 0.0 | 0.1 | 0.1 | 0.0 | 0.0 |
|  | Melanoma of skin | 0.6 | 0.8 | 0.7 | 0.5 | 0.4 |
|  | Breast | 1.2 | 1.7 | 1.4 | 1.0 | 0.7 |
|  | Cervix uteri | 0.3 | 0.5 | 0.3 | 0.1 | 0.2 |
|  | Corpus uteri | 0.0 | 0.1 | 0.0 | 0.0 | 0.0 |
|  | Ovary | 0.2 | 0.3 | 0.2 | 0.2 | 0.1 |
|  | Prostate | 0.0 | 0.0 | 0.0 | 0.0 | 0.0 |
|  | Testis | 0.3 | 0.7 | 0.4 | 0.2 | 0.2 |
|  | Kidney | 0.1 | 0.2 | 0.1 | 0.1 | 0.1 |
|  | Bladder | 0.1 | 0.1 | 0.0 | 0.0 | 0.1 |
|  | Brain and CNS | 1.3 | 1.6 | 1.3 | 1.2 | 1.1 |
|  | Thyroid | 0.0 | 0.0 | 0.0 | 0.0 | 0.0 |
| Hodgkin lymphoma | | 0.4 | 0.9 | 0.4 | 0.2 | 0.1 |
| Non-Hodgkin lymphoma | | 0.5 | 0.8 | 0.7 | 0.4 | 0.3 |
| Multiple myeloma | | 0.0 | 0.1 | 0.0 | 0.0 | 0.0 |
| Leukemia | | 1.1 | 1.7 | 1.2 | 0.9 | 0.6 |
| Other and unspecified malignant neoplasms | | 1.2 | 1.6 | 1.2 | 1.2 | 1.0 |
| Benign or uncertain neoplasms of brain and CNS | | 0.1 | 0.2 | 0.1 | 0.1 | 0.0 |
| Abbreviations: **CNS**, central nervous system | | | | | | |
| ^1^ The total corresponds to the whole of the study period, 1980-2019 | | | | | |  |

**Table S4**. Estimated number of primary cancers (n) and age-standardized incidence (per 100’000 person years and according to the 1976 European standard population) of cancer in female AYAs in Switzerland for different time periods between 1980 and 2019 by language region, age at diagnosis, and cancer category.

| **Population characteristics** | **Estimated number of primary cancers (n)** | | | | | | **Age-standardised incidence** | | | | | | |
| --- | --- | --- | --- | --- | --- | --- | --- | --- | --- | --- | --- | --- | --- |
|  | **Total^1^** | **%** | **1980-1989** | **1990-1999** | **2000-2009** | **2010-2019** | | **Total^1^** | **1980-1989** | **1990-1999** | **2000-2009** | **2010-2019** |  |
| **All cancers** | 37681.1 | 53.9 | 7250.6 | 8829.6 | 9698.3 | 11029.2 | | 71.1 | 59.0 | 65.9 | 71.9 | 80.3 |  |
| **Language region** |  |  |  |  |  |  | |  |  |  |  |  |  |
| German | 26342.4 | 69.9 | 5190.4 | 6309.1 | 6597.0 | 7587.4 | | 70.5 | 59.1 | 66.4 | 69.7 | 79.2 |  |
| French/Italian | 11338.8 | 30.1 | 2060.2 | 2520.6 | 3101.3 | 3441.8 | | 72.7 | 58.8 | 64.8 | 76.9 | 83.0 |  |
| **Age group** |  |  |  |  |  |  | |  |  |  |  |  |  |
| 15-19 | 1753.5 | 4.7 | 339.7 | 376.1 | 458.9 | 535.7 | | 20.3 | 14.2 | 19.0 | 21.7 | 25.1 |  |
| 20-24 | 3219.2 | 8.5 | 676.4 | 750.5 | 809.5 | 909.8 | | 34.4 | 27.0 | 32.9 | 37.2 | 37.9 |  |
| 25-29 | 5797.5 | 15.4 | 1067.1 | 1509.9 | 1333.1 | 1730.5 | | 56.3 | 43.4 | 54.9 | 56.0 | 64.0 |  |
| 30-34 | 10417.3 | 27.7 | 1845.6 | 2506.6 | 2597.6 | 3160.3 | | 95.4 | 75.2 | 86.4 | 95.9 | 110.8 |  |
| 35-39 | 16493.7 | 43.8 | 3321.8 | 3686.5 | 4499.3 | 4692.8 | | 149.3 | 135.4 | 136.2 | 148.7 | 163.9 |  |
| **Cancer category^2,3,4^** |  |  |  |  |  |  | |  |  |  |  |  |  |
| 1. Leukemias and related disorders | 1383.5 | 3.7 | 263.5 | 287.2 | 381.4 | 414.5 | | 2.7 | 2.2 | 2.2 | 3.0 | 3.1 |  |
| 1.1 Acute lymphoblastic leukemia | 224.6 | 16.2 | 49.4 | 52.1 | 51.2 | 67.7 | | 0.5 | 0.4 | 0.4 | 0.4 | 0.5 |  |
| 1.2 Acute myeloid leukemia | 485.4 | 35.1 | 122.4 | 112.4 | 129.3 | 123.0 | | 0.9 | 1.0 | 0.9 | 1.0 | 0.9 |  |
| 1.3 Chronic myeloid leukemia | 226.5 | 16.4 | 49.9 | 53.4 | 63.7 | 60.2 | | 0.4 | 0.4 | 0.4 | 0.5 | 0.4 |  |
| 1.6 Essential thrombocythemia | 138.0 | 10.0 | 4.2 | 21.1 | 29.7 | 64.1 | | 0.3 | 0.0 | 0.2 | 0.2 | 0.5 |  |
| 2. Lymphomas | 3407.4 | 9.0 | 636.2 | 875.5 | 889.8 | 946.2 | | 6.7 | 5.2 | 6.9 | 7.1 | 7.2 |  |
| 2.1 Non-Hodgkin lymphomas | 1322.8 | 38.8 | 214.4 | 384.5 | 350.7 | 356.5 | | 2.5 | 1.7 | 2.9 | 2.7 | 2.6 |  |
| 2.1.2 Burkitt | 62.7 | 4.7 | 7.9 | 31.0 | 16.1 | 10.7 | | 0.1 | 0.1 | 0.2 | 0.1 | 0.1 |  |
| 2.1.3 Diffuse large B-cell (DLBCL) | 510.8 | 38.6 | 82.7 | 161.8 | 143.8 | 124.3 | | 1.0 | 0.7 | 1.2 | 1.1 | 0.9 |  |
| 2.1.5 Anaplastic T-cell and null-cell excluding NK/T-cell | 183.1 | 13.8 | 27.7 | 47.8 | 49.6 | 53.7 | | 0.4 | 0.2 | 0.4 | 0.4 | 0.4 |  |
| 2.1.6 Follicular | 180.7 | 13.7 | 28.7 | 40.5 | 44.8 | 58.0 | | 0.3 | 0.2 | 0.3 | 0.3 | 0.4 |  |
| 2.2 Hodgkin lymphoma | 1870.0 | 54.9 | 359.0 | 427.1 | 492.3 | 542.5 | | 3.8 | 2.9 | 3.5 | 4.1 | 4.3 |  |
| 2.2.1 Hodgkin NLP | 36.3 | 1.9 | 0.0 | 2.2 | 9.6 | 19.1 | | 0.1 | 0.0 | 0.0 | 0.1 | 0.2 |  |
| 2.2.2 Hodgkin classic, other | 1833.7 | 98.1 | 359.0 | 425.0 | 482.7 | 523.4 | | 3.7 | 2.9 | 3.5 | 4.0 | 4.1 |  |
| 3. CNS and other intracranial and intraspinal neoplasms | 2801.5 | 7.4 | 450.5 | 518.2 | 717.2 | 954.1 | | 5.4 | 3.7 | 4.0 | 5.5 | 7.1 |  |
| 3.1 Astroglial and related neoplasms | 1185.4 | 42.3 | 240.9 | 259.2 | 336.4 | 329.8 | | 2.3 | 2.0 | 2.0 | 2.6 | 2.5 |  |
| 3.1.1 Oligodendriogliomas | 240.3 | 20.3 | 31.6 | 51.3 | 74.3 | 74.3 | | 0.5 | 0.3 | 0.4 | 0.6 | 0.6 |  |
| 3.1.2 Glioblastomas/gliofibromas | 193.4 | 16.3 | 31.9 | 51.4 | 60.2 | 49.6 | | 0.4 | 0.3 | 0.4 | 0.5 | 0.4 |  |
| 3.1.3 Ependymomas | 160.8 | 13.6 | 25.8 | 7.7 | 46.7 | 64.0 | | 0.3 | 0.2 | 0.1 | 0.4 | 0.5 |  |
| 3.4 Neuronal and mixed neuronal-glial neoplasms | 119.9 | 4.3 | 21.3 | 11.9 | 34.0 | 44.8 | | 0.2 | 0.2 | 0.1 | 0.3 | 0.4 |  |
| 3.4.1 Neuronal and mixed neuronal-glial, benign/borderline | 119.9 | 100.0 | 21.3 | 11.9 | 34.0 | 44.8 | | 0.2 | 0.2 | 0.1 | 0.3 | 0.4 |  |
| 3.5 Meningiomas | 754.3 | 26.9 | 64.9 | 135.6 | 171.3 | 307.3 | | 1.4 | 0.5 | 1.0 | 1.2 | 2.2 |  |
| 3.5.1 Meningioma, benign/borderline | 731.3 | 97.0 | 58.2 | 127.9 | 168.2 | 301.5 | | 1.4 | 0.5 | 0.9 | 1.2 | 2.2 |  |
| 3.8 Pituitary neoplasms | 438.5 | 15.7 | 54.3 | 69.3 | 100.7 | 174.8 | | 0.9 | 0.4 | 0.5 | 0.8 | 1.3 |  |
| 3.8.1 Pituitary, benign/borderline | 426.4 | 97.2 | 46.4 | 65.9 | 100.7 | 172.5 | | 0.8 | 0.4 | 0.5 | 0.8 | 1.3 |  |
| 4. Sarcomas | 1363.9 | 3.6 | 299.6 | 342.5 | 326.8 | 379.2 | | 2.7 | 2.4 | 2.7 | 2.6 | 2.9 |  |
| 4.1 Osteosarcoma | 136.0 | 10.0 | 21.2 | 49.7 | 31.7 | 34.4 | | 0.3 | 0.2 | 0.4 | 0.3 | 0.3 |  |
| 4.2 Chondrosarcoma | 134.7 | 9.9 | 19.0 | 38.4 | 34.8 | 39.1 | | 0.3 | 0.2 | 0.3 | 0.3 | 0.3 |  |
| 4.3 Ewing family of tumors | 127.4 | 9.3 | 28.1 | 19.4 | 21.1 | 49.4 | | 0.3 | 0.2 | 0.2 | 0.2 | 0.4 |  |
| 4.4 Fibromatous neoplasms | 291.2 | 21.4 | 67.6 | 101.6 | 54.2 | 71.5 | | 0.6 | 0.6 | 0.8 | 0.4 | 0.5 |  |
| 4.5 Liposarcoma | 98.4 | 7.2 | 23.9 | 12.5 | 29.8 | 29.4 | | 0.2 | 0.2 | 0.1 | 0.2 | 0.2 |  |
| 5. Blood and lymphatic vessel tumor | 216.9 | 0.6 | 43.9 | 53.6 | 72.0 | 48.6 | | 0.4 | 0.4 | 0.4 | 0.6 | 0.4 |  |
| 5.2 Malignant blood and lymphatic vessel tumors, all sites | 132.6 | 61.1 | 24.0 | 39.6 | 39.6 | 30.7 | | 0.3 | 0.2 | 0.3 | 0.3 | 0.2 |  |
| 5.2.1 Kaposi sarcoma | 60.1 | 45.3 | 5.9 | 26.3 | 24.6 | 6.4 | | 0.1 | 0.1 | 0.2 | 0.2 | 0.1 |  |
| 6. Nerve sheath tumors | 291.9 | 0.8 | 34.9 | 61.6 | 60.1 | 113.9 | | 0.6 | 0.3 | 0.5 | 0.5 | 0.8 |  |
| 6.1 Benign, CNS | 256.1 | 87.7 | 31.0 | 51.7 | 48.4 | 103.5 | | 0.5 | 0.3 | 0.4 | 0.4 | 0.7 |  |
| 6.1.1 Neurilemmoma | 240.3 | 93.8 | 26.4 | 49.1 | 42.1 | 100.1 | | 0.5 | 0.2 | 0.4 | 0.3 | 0.7 |  |
| 7. Gonadal and related tumors | 1236.1 | 3.3 | 341.4 | 356.5 | 267.4 | 293.4 | | 2.4 | 2.8 | 2.7 | 2.0 | 2.2 |  |
| 7.2 Ovary | 1110.4 | 89.8 | 307.8 | 328.9 | 232.5 | 262.1 | | 2.1 | 2.5 | 2.5 | 1.7 | 2.0 |  |
| 7.2.1 Germ cell and trophoblastic | 288.0 | 25.9 | 67.7 | 76.4 | 49.4 | 88.1 | | 0.6 | 0.6 | 0.6 | 0.4 | 0.7 |  |
| 7.2.2 Non-germ cell | 822.4 | 74.1 | 240.1 | 252.5 | 183.1 | 174.0 | | 1.5 | 2.0 | 1.9 | 1.3 | 1.3 |  |
| 7.4 Germ cell and trophoblastic excluding CNS, ovary, testis | 109.9 | 8.9 | 29.7 | 24.5 | 31.6 | 26.5 | | 0.2 | 0.2 | 0.2 | 0.3 | 0.2 |  |
| 8. Melanoma, malignant | 5998.6 | 15.9 | 951.2 | 1544.0 | 1776.9 | 1608.2 | | 11.4 | 7.7 | 11.6 | 13.5 | 11.6 |  |
| 8.1 Superficial spreading/low cumulative sun damage melanoma | 3675.6 | 61.3 | 466.7 | 966.3 | 1077.1 | 1037.2 | | 6.9 | 3.8 | 7.2 | 8.2 | 7.5 |  |
| 8.2 Nodular melanoma | 460.8 | 7.7 | 168.9 | 145.2 | 124.9 | 63.1 | | 0.9 | 1.4 | 1.1 | 1.0 | 0.5 |  |
| 9. Carcinomas | 20706.9 | 55.0 | 4146.1 | 4719.0 | 5132.3 | 6216.0 | | 38.4 | 33.8 | 34.5 | 36.7 | 44.6 |  |
| 9.1 Thyroid carcinoma | 3653.1 | 17.6 | 433.0 | 680.9 | 961.9 | 1338.9 | | 6.9 | 3.5 | 5.1 | 7.2 | 9.8 |  |
| 9.1.3 Papillary | 2218.2 | 60.7 | 198.0 | 300.3 | 628.5 | 887.6 | | 4.2 | 1.6 | 2.3 | 4.7 | 6.5 |  |
| 9.1.4 Follicular | 465.7 | 12.7 | 60.8 | 115.0 | 141.2 | 139.6 | | 0.9 | 0.5 | 0.8 | 1.1 | 1.0 |  |
| 9.1.5 Papillary with follicular variant | 813.2 | 22.3 | 133.3 | 210.8 | 164.7 | 273.3 | | 1.5 | 1.1 | 1.6 | 1.2 | 2.0 |  |
| 9.2 Other carcinoma of head and neck | 487.0 | 2.4 | 82.1 | 128.9 | 118.9 | 138.3 | | 0.9 | 0.7 | 1.0 | 0.9 | 1.0 |  |
| 9.2.2 Oral cavity, lip, and pharynx | 237.1 | 48.7 | 39.1 | 51.2 | 70.8 | 65.6 | | 0.4 | 0.3 | 0.4 | 0.5 | 0.5 |  |
| 9.2.3 Salivary gland | 122.4 | 25.1 | 13.1 | 38.9 | 22.4 | 40.7 | | 0.2 | 0.1 | 0.3 | 0.2 | 0.3 |  |
| 9.3 Carcinoma of gastrointestinal tract | 2484.9 | 12.0 | 433.4 | 546.6 | 527.2 | 860.1 | | 4.7 | 3.5 | 4.0 | 3.8 | 6.4 |  |
| 9.3.2 Carcinoma of stomach | 419.6 | 16.9 | 112.0 | 108.4 | 96.9 | 107.8 | | 0.8 | 0.9 | 0.8 | 0.7 | 0.8 |  |
| 9.3.4 Carcinoma of colon | 1105.6 | 44.5 | 157.7 | 198.3 | 214.9 | 443.4 | | 2.1 | 1.3 | 1.5 | 1.6 | 3.4 |  |
| 9.3.5 Carcinoma of rectum | 465.1 | 18.7 | 79.6 | 116.7 | 95.4 | 154.1 | | 0.9 | 0.7 | 0.9 | 0.7 | 1.1 |  |
| 9.3.7 Carcinoma of liver and intrahepatic bile ducts (IBD) | 94.6 | 3.8 | 21.5 | 25.9 | 21.3 | 26.3 | | 0.2 | 0.2 | 0.2 | 0.2 | 0.2 |  |
| 9.3.9 Carcinoma of pancreas | 159.5 | 6.4 | 25.0 | 30.5 | 36.7 | 59.8 | | 0.3 | 0.2 | 0.2 | 0.3 | 0.5 |  |
| 9.4 Carcinoma of lung, bronchus, and trachea | 544.4 | 2.6 | 136.8 | 165.7 | 123.8 | 127.1 | | 1.0 | 1.1 | 1.2 | 0.9 | 0.9 |  |
| 9.4.2 Non-small cell carcinoma | 507.4 | 93.2 | 124.5 | 146.4 | 116.2 | 124.8 | | 0.9 | 1.0 | 1.1 | 0.9 | 0.9 |  |
| 9.6 Carcinoma of breast | 9611.7 | 46.4 | 1980.5 | 2030.2 | 2463.6 | 2889.2 | | 17.6 | 16.1 | 14.7 | 17.2 | 20.4 |  |
| 9.6.1 Breast, infiltrating duct | 8036.0 | 83.6 | 1346.3 | 1663.9 | 2121.7 | 2570.6 | | 14.7 | 11.0 | 12.1 | 14.8 | 18.1 |  |
| 9.6.2 Breast, adenocarcinoma | 469.8 | 4.9 | 306.1 | 59.4 | 65.2 | 83.9 | | 0.9 | 2.5 | 0.4 | 0.5 | 0.6 |  |
| 9.6.3 Breast, lobular | 435.8 | 4.5 | 116.2 | 127.4 | 125.3 | 84.1 | | 0.8 | 1.0 | 0.9 | 0.9 | 0.6 |  |
| 9.6.5 Breast, medullary | 253.2 | 2.6 | 84.8 | 67.2 | 69.3 | 47.4 | | 0.5 | 0.7 | 0.5 | 0.5 | 0.3 |  |
| 9.7 Carcinoma of genital sites excluding ovary and testis | 3338.3 | 16.1 | 954.5 | 1002.4 | 781.9 | 715.9 | | 6.2 | 7.8 | 7.3 | 5.5 | 5.1 |  |
| 9.7.1 Carcinoma of uterine cervix | 2791.6 | 83.6 | 844.3 | 859.5 | 611.8 | 586.2 | | 5.2 | 6.9 | 6.2 | 4.4 | 4.2 |  |
| 9.7.2 Corpus uteri | 407.3 | 12.2 | 72.2 | 98.1 | 126.1 | 108.4 | | 0.7 | 0.6 | 0.7 | 0.9 | 0.8 |  |
| 9.8 Carcinoma of urinary tract | 334.2 | 1.6 | 71.7 | 83.7 | 84.6 | 89.7 | | 0.6 | 0.6 | 0.6 | 0.6 | 0.6 |  |
| 9.8.1 Carcinoma of kidney | 227.7 | 68.1 | 43.8 | 47.5 | 68.6 | 64.3 | | 0.4 | 0.4 | 0.4 | 0.5 | 0.5 |  |
| 9.8.2 Carcinoma of bladder | 95.0 | 28.4 | 25.6 | 31.9 | 15.0 | 21.9 | | 0.2 | 0.2 | 0.2 | 0.1 | 0.2 |  |
| 10. Miscellaneous specified neoplasms | 116.8 | 0.3 | 38.9 | 28.9 | 37.9 | 18.8 | | 0.2 | 0.3 | 0.2 | 0.3 | 0.1 |  |
| 11. Unspecified malignant neoplasms except CNS | 157.7 | 0.4 | 44.5 | 42.7 | 36.6 | 36.4 | | 0.3 | 0.4 | 0.3 | 0.3 | 0.3 |  |

Abbreviations: **CNS**, central nervous system

^1^ The total corresponds to the whole of the study period, 1980-2019

^2^ Cancer categories according to Barr et al. [2]

^3^ Cancer categories reported only if included in Table 1, excluding cance e.g. 7.1. Testis

^4^ Proportions (%) are reported within each upper level, e.g. the percentage of all level 1 cancer categories are calculated over the total number of primary cancers, whereas the percentage of, for example, acute lymphoblastic leukemias (category 1.1) is calculated over all leukemias and related disorders (category 1)

**Table S5**. Estimated number of primary cancers (n) and age-standardized incidence (per 100’000 person years and according to the 1976 European standard population) cancer in male AYAs in Switzerland for different time periods between 1980 and 2019 by language region, age at diagnosis, and cancer category.

| **Population characteristics** | **Estimated number of primary cancers (n)** | | | | | | | **Age-standardised incidence** | | | | | |
| --- | --- | --- | --- | --- | --- | --- | --- | --- | --- | --- | --- | --- | --- |
|  | **Total^1^** | **%** | **1980-1989** | **1990-1999** | **2000-2009** | **2010-2019** | **Total^1^** | | **1980-1989** | **1990-1999** | **2000-2009** | **2010-2019** |  |
| **All cancers** | 32219.7 | 46.1 | 6795.8 | 8300.9 | 7966.3 | 8844.8 | 60.9 | | 54.4 | 61.6 | 60.7 | 64.1 |  |
| **Language region** |  |  |  |  |  |  |  | |  |  |  |  |  |
| German | 22963.6 | 71.3 | 4927.7 | 5970.5 | 5611.9 | 6215.2 | 60.9 | | 54.3 | 61.9 | 60.5 | 64.1 |  |
| French/Italian | 9256.1 | 28.7 | 1868.1 | 2330.4 | 2354.5 | 2629.7 | 60.9 | | 54.6 | 60.6 | 61.3 | 64.1 |  |
| **Age group** |  |  |  |  |  |  |  | |  |  |  |  |  |
| 15-19 | 2119.7 | 6.6 | 437.6 | 487.7 | 515.4 | 639.9 | 23.3 | | 17.4 | 23.4 | 23.2 | 28.3 |  |
| 20-24 | 4236.8 | 13.2 | 1038.7 | 982.7 | 1032.2 | 1147.2 | 44.4 | | 40.9 | 42.8 | 46.7 | 45.9 |  |
| 25-29 | 6262.2 | 19.4 | 1422.0 | 1664.7 | 1421.8 | 1716.2 | 60.9 | | 58.3 | 61.2 | 60.4 | 62.1 |  |
| 30-34 | 8635.6 | 26.8 | 1736.1 | 2364.0 | 2064.7 | 2386.8 | 78.4 | | 69.9 | 80.2 | 77.1 | 82.4 |  |
| 35-39 | 10965.4 | 34.0 | 2161.4 | 2801.8 | 2932.2 | 2954.8 | 97.3 | | 85.4 | 100.3 | 96.4 | 101.8 |  |
| **Cancer category^2,3,4^** |  |  |  |  |  |  |  | |  |  |  |  |  |
| 1. Leukemias and related disorders | 1894.7 | 5.9 | 369.1 | 454.4 | 511.5 | 537.0 | 3.6 | | 3.0 | 3.5 | 4.0 | 4.0 |  |
| 1.1 Acute lymphoblastic leukemia | 425.8 | 22.5 | 98.7 | 103.7 | 107.6 | 111.3 | 0.9 | | 0.8 | 0.9 | 0.9 | 0.9 |  |
| 1.2 Acute myeloid leukemia | 566.4 | 29.9 | 126.9 | 165.6 | 150.8 | 132.4 | 1.1 | | 1.0 | 1.3 | 1.2 | 1.0 |  |
| 1.3 Chronic myeloid leukemia | 342.6 | 18.1 | 66.0 | 99.5 | 98.3 | 83.5 | 0.7 | | 0.5 | 0.7 | 0.7 | 0.6 |  |
| 1.6 Essential thrombocythemia | 111.6 | 5.9 | 8.0 | 6.9 | 28.3 | 54.7 | 0.2 | | 0.1 | 0.1 | 0.2 | 0.4 |  |
| 2. Lymphomas | 4800.8 | 14.9 | 1013.1 | 1286.6 | 1208.3 | 1293.1 | 9.2 | | 8.1 | 9.7 | 9.5 | 9.5 |  |
| 2.1 Non-Hodgkin lymphomas | 2061.8 | 42.9 | 392.9 | 636.1 | 543.6 | 506.9 | 3.9 | | 3.1 | 4.7 | 4.1 | 3.7 |  |
| 2.1.2 Burkitt | 189.5 | 9.2 | 39.7 | 46.9 | 56.8 | 44.5 | 0.4 | | 0.3 | 0.4 | 0.4 | 0.3 |  |
| 2.1.3 Diffuse large B-cell (DLBCL) | 801.5 | 38.9 | 139.0 | 315.8 | 214.9 | 160.5 | 1.5 | | 1.1 | 2.3 | 1.6 | 1.2 |  |
| 2.1.5 Anaplastic T-cell and null-cell excluding NK/T-cell | 295.2 | 14.3 | 43.1 | 63.4 | 85.8 | 91.8 | 0.6 | | 0.4 | 0.5 | 0.7 | 0.7 |  |
| 2.1.6 Follicular | 240.3 | 11.7 | 40.3 | 58.0 | 72.9 | 67.5 | 0.4 | | 0.3 | 0.4 | 0.5 | 0.5 |  |
| 2.2 Hodgkin lymphoma | 2373.1 | 49.4 | 487.7 | 545.9 | 606.0 | 701.0 | 4.6 | | 3.9 | 4.2 | 4.9 | 5.3 |  |
| 2.2.2 Hodgkin classic, other | 2226.2 | 93.8 | 487.7 | 539.8 | 557.3 | 629.9 | 4.4 | | 3.9 | 4.2 | 4.5 | 4.7 |  |
| 3. CNS and other intracranial and intraspinal neoplasms | 2609.1 | 8.1 | 493.9 | 546.5 | 615.1 | 835.4 | 5.0 | | 4.0 | 4.1 | 4.7 | 6.1 |  |
| 3.1 Astroglial and related neoplasms | 1640.2 | 62.9 | 344.0 | 389.9 | 386.4 | 479.8 | 3.1 | | 2.8 | 3.0 | 3.0 | 3.5 |  |
| 3.1.1 Oligodendriogliomas | 323.9 | 19.7 | 52.5 | 63.7 | 91.4 | 101.9 | 0.6 | | 0.4 | 0.5 | 0.7 | 0.7 |  |
| 3.1.2 Glioblastomas/gliofibromas | 277.7 | 16.9 | 43.1 | 55.0 | 77.1 | 88.0 | 0.5 | | 0.4 | 0.4 | 0.6 | 0.6 |  |
| 3.1.3 Ependymomas | 220.6 | 13.5 | 35.2 | 39.3 | 45.3 | 83.1 | 0.4 | | 0.3 | 0.3 | 0.4 | 0.6 |  |
| 3.4 Neuronal and mixed neuronal-glial neoplasms | 139.4 | 5.3 | 9.9 | 18.3 | 40.7 | 57.7 | 0.3 | | 0.1 | 0.2 | 0.3 | 0.5 |  |
| 3.4.1 Neuronal and mixed neuronal-glial, benign/borderline | 132.7 | 95.2 | 7.6 | 14.0 | 39.6 | 57.7 | 0.3 | | 0.1 | 0.1 | 0.3 | 0.5 |  |
| 3.5 Meningiomas | 330.1 | 12.7 | 54.2 | 52.3 | 78.6 | 119.1 | 0.6 | | 0.4 | 0.4 | 0.6 | 0.9 |  |
| 3.5.1 Meningioma, benign/borderline | 311.9 | 94.5 | 49.6 | 45.8 | 75.4 | 114.5 | 0.6 | | 0.4 | 0.3 | 0.5 | 0.8 |  |
| 3.8 Pituitary neoplasms | 223.9 | 8.6 | 31.1 | 22.0 | 51.9 | 94.0 | 0.4 | | 0.3 | 0.2 | 0.4 | 0.7 |  |
| 3.8.1 Pituitary, benign/borderline | 214.2 | 95.7 | 21.8 | 19.5 | 51.9 | 94.0 | 0.4 | | 0.2 | 0.2 | 0.4 | 0.7 |  |
| 4. Sarcomas | 1551.3 | 4.8 | 343.3 | 411.4 | 384.6 | 411.9 | 3.0 | | 2.7 | 3.2 | 3.0 | 3.1 |  |
| 4.1 Osteosarcoma | 211.8 | 13.7 | 56.8 | 47.3 | 55.1 | 54.1 | 0.4 | | 0.5 | 0.4 | 0.5 | 0.4 |  |
| 4.2 Chondrosarcoma | 159.7 | 10.3 | 27.2 | 41.2 | 37.8 | 47.4 | 0.3 | | 0.2 | 0.3 | 0.3 | 0.4 |  |
| 4.3 Ewing family of tumors | 172.5 | 11.1 | 38.3 | 46.6 | 35.3 | 49.7 | 0.4 | | 0.3 | 0.4 | 0.3 | 0.4 |  |
| 4.4 Fibromatous neoplasms | 298.7 | 19.3 | 75.7 | 102.0 | 78.9 | 57.2 | 0.6 | | 0.6 | 0.8 | 0.6 | 0.4 |  |
| 4.5 Liposarcoma | 160.9 | 10.4 | 36.5 | 35.5 | 50.5 | 38.1 | 0.3 | | 0.3 | 0.3 | 0.4 | 0.3 |  |
| 5. Blood and lymphatic vessel tumor | 856.8 | 2.7 | 259.8 | 485.6 | 120.4 | 85.4 | 1.6 | | 2.1 | 3.5 | 0.9 | 0.6 |  |
| 5.2 Malignant blood and lymphatic vessel tumors, all sites | 767.0 | 89.5 | 246.6 | 477.8 | 93.6 | 50.5 | 1.4 | | 2.0 | 3.4 | 0.7 | 0.4 |  |
| 5.2.1 Kaposi sarcoma | 703.9 | 91.8 | 227.9 | 460.0 | 76.7 | 37.7 | 1.3 | | 1.8 | 3.3 | 0.6 | 0.3 |  |
| 6. Nerve sheath tumors | 323.1 | 1.0 | 40.9 | 72.0 | 75.5 | 114.5 | 0.6 | | 0.3 | 0.6 | 0.6 | 0.8 |  |
| 6.1 Benign, CNS | 243.2 | 75.3 | 26.4 | 47.2 | 60.7 | 89.5 | 0.5 | | 0.2 | 0.4 | 0.4 | 0.6 |  |
| 6.1.1 Neurilemmoma | 232.3 | 95.6 | 26.4 | 43.3 | 60.7 | 83.8 | 0.4 | | 0.2 | 0.3 | 0.4 | 0.6 |  |
| 7. Gonadal and related tumors | 10066.5 | 31.2 | 2257.5 | 2539.9 | 2498.5 | 2707.3 | 19.1 | | 18.1 | 18.8 | 19.3 | 19.6 |  |
| 7.1 Testis | 9781.1 | 97.2 | 2171.7 | 2461.4 | 2443.1 | 2634.0 | 18.5 | | 17.4 | 18.2 | 18.9 | 19.0 |  |
| 7.1.1 Germ cell and trophoblastic | 9748.9 | 99.7 | 2167.8 | 2451.0 | 2431.6 | 2626.9 | 18.5 | | 17.4 | 18.1 | 18.8 | 19.0 |  |
| 7.4 Germ cell and trophoblastic excluding CNS, ovary, testis | 213.6 | 2.1 | 74.6 | 61.8 | 41.1 | 47.4 | 0.4 | | 0.6 | 0.5 | 0.3 | 0.4 |  |
| 8. Melanoma, malignant | 3696.4 | 11.5 | 555.4 | 959.5 | 1094.9 | 1001.5 | 6.9 | | 4.4 | 7.1 | 8.2 | 7.1 |  |
| 8.1 Superficial spreading/low cumulative sun damage melanoma | 2117.3 | 57.3 | 275.3 | 527.3 | 651.9 | 593.5 | 3.9 | | 2.2 | 3.9 | 4.8 | 4.2 |  |
| 8.2 Nodular melanoma | 403.4 | 10.9 | 104.8 | 137.0 | 109.3 | 71.9 | 0.8 | | 0.8 | 1.0 | 0.8 | 0.5 |  |
| 9. Carcinomas | 6194.7 | 19.2 | 1384.1 | 1478.9 | 1411.3 | 1812.8 | 11.4 | | 11.0 | 10.7 | 10.3 | 12.9 |  |
| 9.1 Thyroid carcinoma | 1131.8 | 18.3 | 139.5 | 228.5 | 277.6 | 407.1 | 2.1 | | 1.1 | 1.7 | 2.1 | 2.9 |  |
| 9.1.3 Papillary | 660.9 | 58.4 | 40.2 | 94.5 | 162.0 | 285.4 | 1.2 | | 0.3 | 0.7 | 1.2 | 2.1 |  |
| 9.1.4 Follicular | 140.9 | 12.4 | 21.6 | 42.1 | 39.7 | 37.3 | 0.3 | | 0.2 | 0.3 | 0.3 | 0.3 |  |
| 9.1.5 Papillary with follicular variant | 240.1 | 21.2 | 45.7 | 63.7 | 53.4 | 71.6 | 0.5 | | 0.4 | 0.5 | 0.4 | 0.5 |  |
| 9.2 Other carcinoma of head and neck | 882.0 | 14.2 | 257.5 | 247.7 | 215.4 | 189.5 | 1.6 | | 2.1 | 1.8 | 1.6 | 1.3 |  |
| 9.2.2 Oral cavity, lip, and pharynx | 469.9 | 53.3 | 160.2 | 127.1 | 98.9 | 102.4 | 0.9 | | 1.3 | 0.9 | 0.7 | 0.7 |  |
| 9.2.3 Salivary gland | 126.9 | 14.39 | 18.0 | 44.9 | 35.0 | 29.0 | 0.2 | | 0.1 | 0.3 | 0.3 | 0.2 |  |
| 9.3 Carcinoma of gastrointestinal tract | 2548.6 | 41.1 | 524.0 | 602.1 | 563.5 | 787.1 | 4.7 | | 4.2 | 4.3 | 4.1 | 5.6 |  |
| 9.3.2 Carcinoma of stomach | 469.1 | 18.4 | 113.4 | 144.0 | 114.9 | 105.1 | 0.9 | | 0.9 | 1.0 | 0.8 | 0.7 |  |
| 9.3.4 Carcinoma of colon | 968.5 | 38.0 | 172.8 | 175.9 | 189.5 | 365.5 | 1.8 | | 1.4 | 1.3 | 1.4 | 2.6 |  |
| 9.3.5 Carcinoma of rectum | 469.9 | 18.4 | 106.7 | 106.8 | 105.1 | 142.2 | 0.9 | | 0.9 | 0.8 | 0.7 | 1.0 |  |
| 9.3.7 Carcinoma of liver and intrahepatic bile ducts (IBD) | 175.4 | 6.9 | 27.4 | 47.3 | 52.5 | 46.6 | 0.3 | | 0.2 | 0.4 | 0.4 | 0.3 |  |
| 9.3.9 Carcinoma of pancreas | 191.0 | 7.5 | 36.6 | 60.2 | 35.9 | 53.7 | 0.4 | | 0.3 | 0.4 | 0.3 | 0.4 |  |
| 9.4 Carcinoma of lung, bronchus, and trachea | 682.8 | 11.0 | 221.6 | 190.1 | 140.9 | 155.8 | 1.2 | | 1.8 | 1.4 | 1.0 | 1.1 |  |
| 9.4.2 Non-small cell carcinoma | 603.2 | 88.3 | 176.3 | 162.8 | 125.0 | 151.1 | 1.1 | | 1.4 | 1.2 | 0.9 | 1.1 |  |
| 9.6 Carcinoma of breast | 17.6 | 0.3 | 4.0 | 0.0 | 4.3 | 7.2 | 0.0 | | 0.0 | 0.0 | 0.0 | 0.1 |  |
| 9.6.1 Breast, infiltrating duct | 9.7 | 55.2 | 0.0 | 0.0 | 1.1 | 6.0 | 0.0 | | 0.0 | 0.0 | 0.0 | 0.0 |  |
| 9.6.2 Breast, adenocarcinoma | 6.7 | 37.9 | 4.0 | 0.0 | 2.1 | 1.2 | 0.0 | | 0.0 | 0.0 | 0.0 | 0.0 |  |
| 9.6.3 Breast, lobular | 1.2 | 6.9 | 0.0 | 0.0 | 1.1 | 0.0 | 0.0 | | 0.0 | 0.0 | 0.0 | 0.0 |  |
| 9.6.5 Breast, medullary | 0.0 | 0.0 | 0.0 | 0.0 | 0.0 | 0.0 | 0.0 | | 0.0 | 0.0 | 0.0 | 0.0 |  |
| 9.7 Carcinoma of genital sites excluding ovary and testis | 66.8 | 1.1 | 15.8 | 9.6 | 27.8 | 13.2 | 0.1 | | 0.1 | 0.1 | 0.2 | 0.1 |  |
| 9.8 Carcinoma of urinary tract | 675.6 | 10.9 | 162.7 | 147.5 | 157.5 | 198.5 | 1.2 | | 1.3 | 1.1 | 1.1 | 1.4 |  |
| 9.8.1 Carcinoma of kidney | 474.2 | 70.2 | 64.2 | 92.2 | 117.3 | 169.3 | 0.9 | | 0.5 | 0.7 | 0.9 | 1.2 |  |
| 9.8.2 Carcinoma of bladder | 169.2 | 25.0 | 85.1 | 49.8 | 30.8 | 23.2 | 0.3 | | 0.7 | 0.4 | 0.2 | 0.2 |  |
| 10. Miscellaneous specified neoplasms | 119.7 | 0.4 | 37.2 | 36.6 | 33.5 | 20.3 | 0.2 | | 0.3 | 0.3 | 0.3 | 0.2 |  |
| 11. Unspecified malignant neoplasms except CNS | 106.8 | 0.3 | 41.5 | 29.6 | 12.8 | 25.7 | 0.2 | | 0.3 | 0.2 | 0.1 | 0.2 |  |
|  | | | | | | | | | | | | | |

Abbreviations: **CNS**, central nervous system

^1^ The total corresponds to the whole of the study period, 1980-2019

^2^ Cancer categories according to Barr et al. [2]

^3^ Cancer categories reported only if included in Table 1, excluding cancers diagnosed only to females, e.g. 7.2 Ovary

^4^ Proportions (%) are reported within each upper level, e.g. the percentage of all level 1 cancer categories are calculated over the total number of primary cancers, whereas the percentage of, for example, acute lymphoblastic leukemias (category 1.1) is calculated over all leukemias and related disorders (category 1)

| **(a)** | **(b)** |
| --- | --- |
| 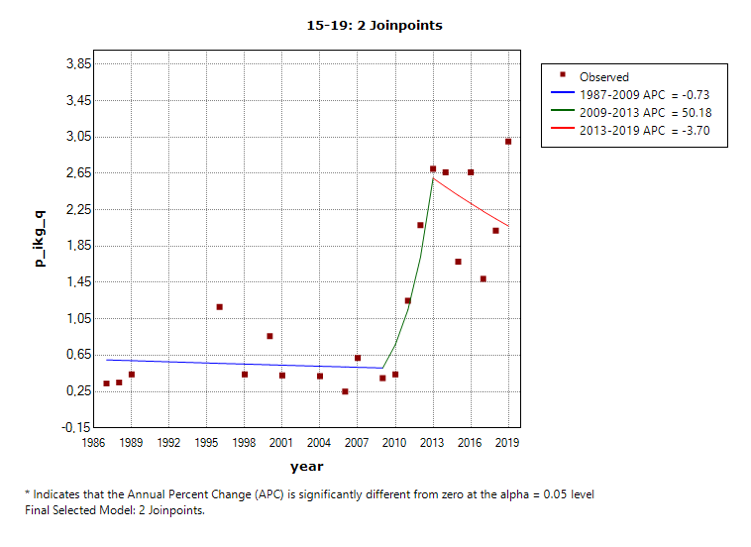 | 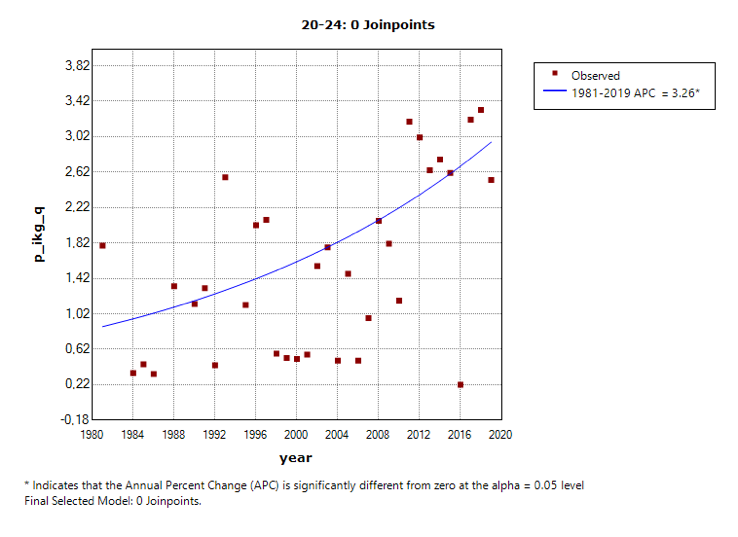 |
| **(c)** | **(d)** |
| 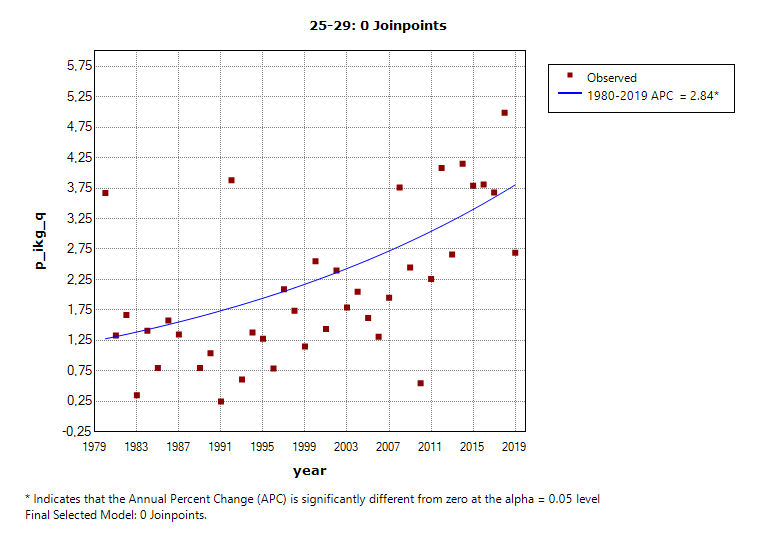 | 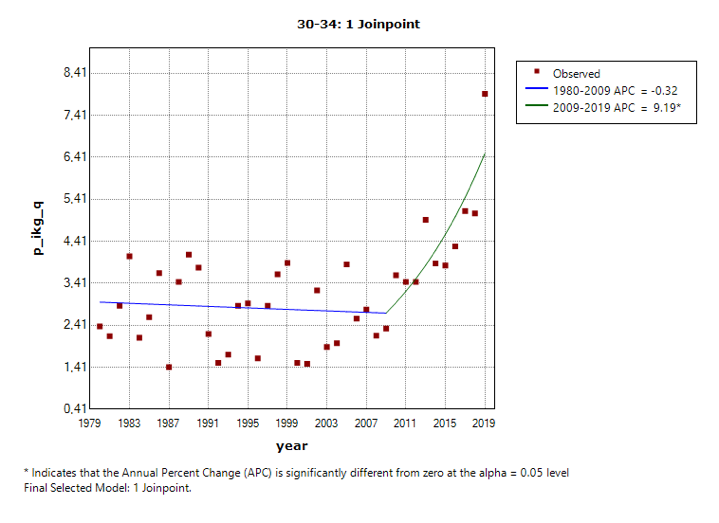 |
| **(e)** |  |
| 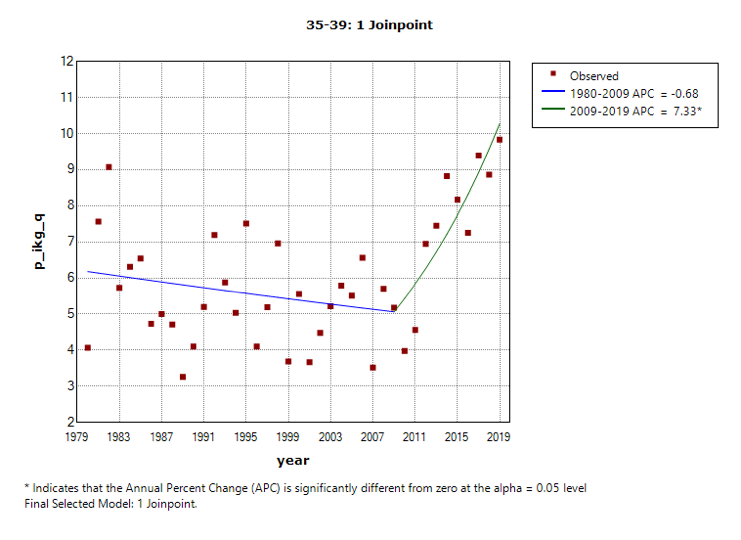 |  |

Figure S1. Trends in age-specific incidence per 100’000 person years for colorectal carcinoma (cancer category numbers 9.3.4-9.3.5 from Barr et al. [2]) in Switzerland between 1980 and 2019. Figure as returned from the Joinpoint software (p_ikg_q, age-specific incidence for the age groups (a) 15-19, (b) 20-24, (c) 25-29, (d) 30-34, and (e) 35-39 years).


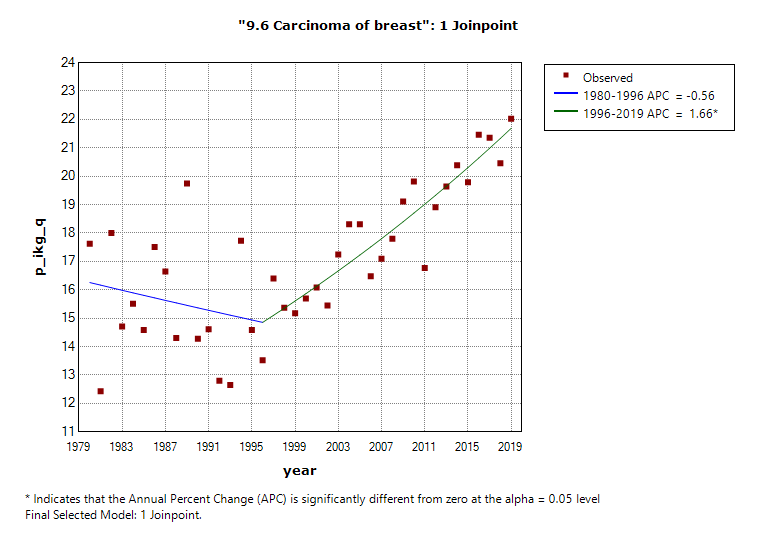


Figure S2. Trends in age-standardized incidence per 100’000 person years for female breast carcinoma in Switzerland between 1980 and 2019. Figure as returned from the Joinpoint software (p_ikg_q, age-standardized incidence).

| 1. **Females** |
| --- |
| 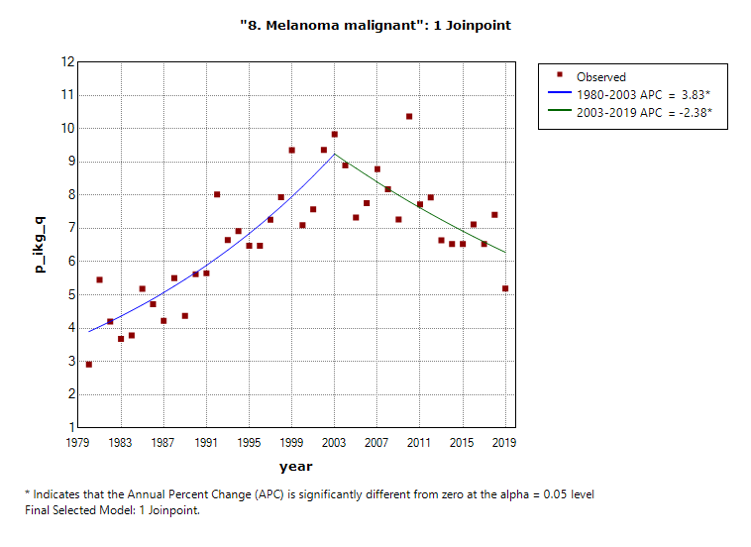 |
|  |
| 1. **Males** |
| 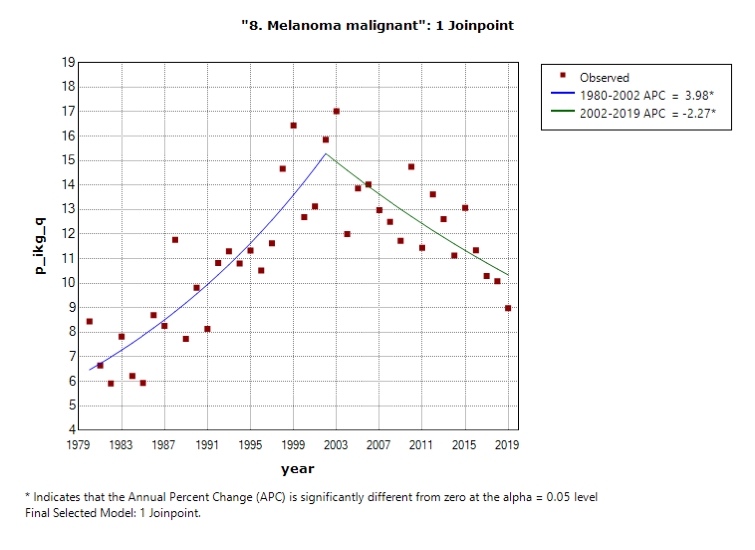 |

Figure S3. Trends in age-standardized incidence per 100’000 person years for malignant melanoma in females (a) and in males (b) in Switzerland between 1980 and 2019. Figure as returned from the Joinpoint software (p_ikg_q, age-standardized incidence).

| **(a)** | **(b)** |
| --- | --- |
| 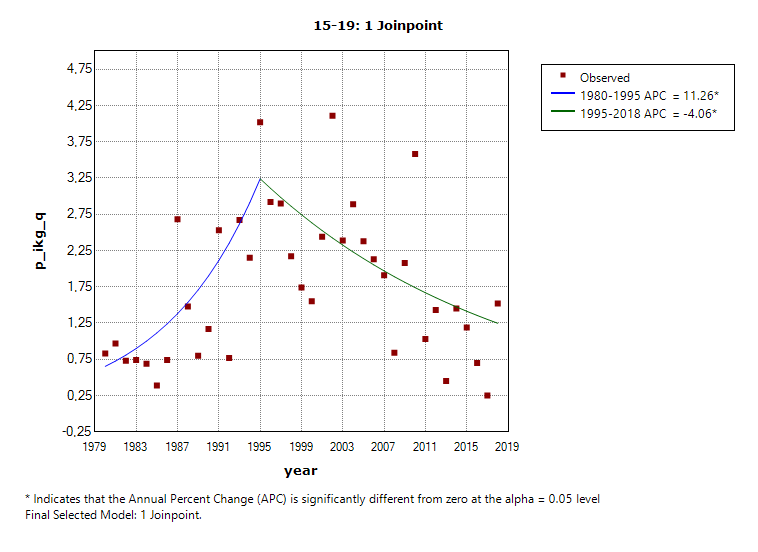 | 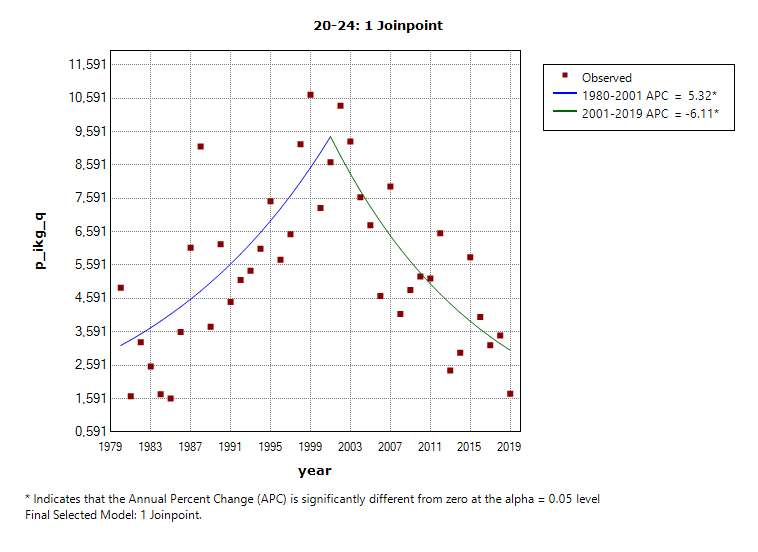 |
| **(c)** | **(d)** |
| 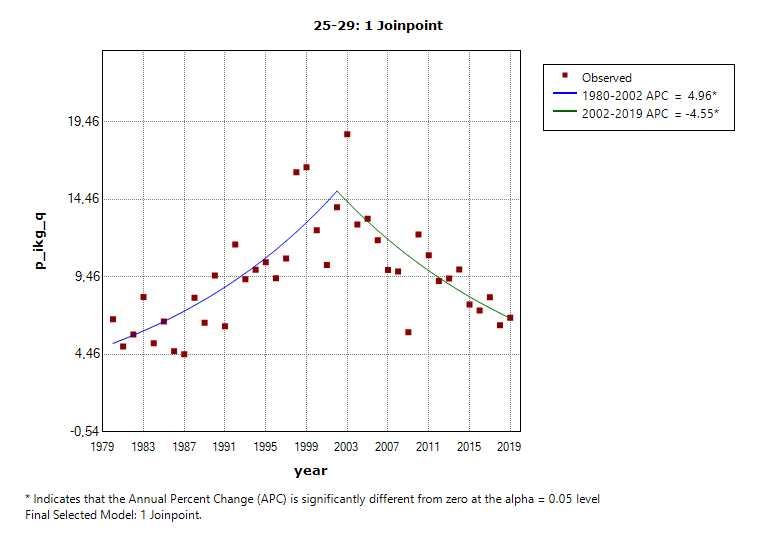 | 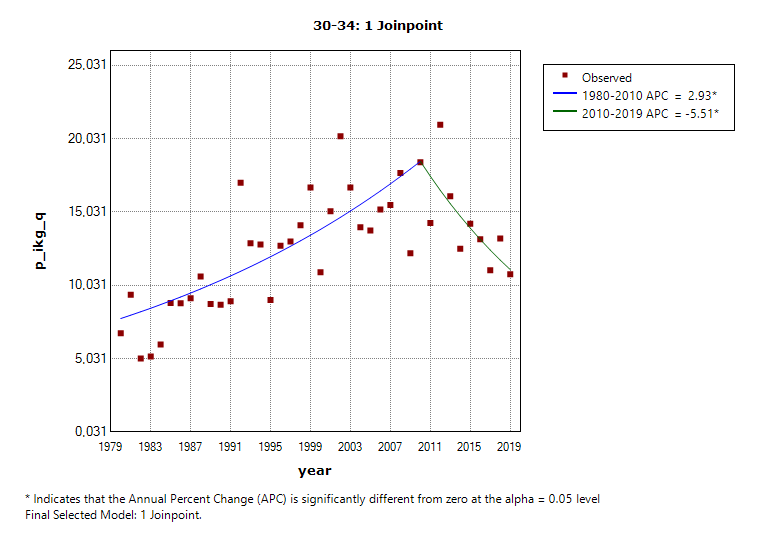 |
| **(e)** |  |
| 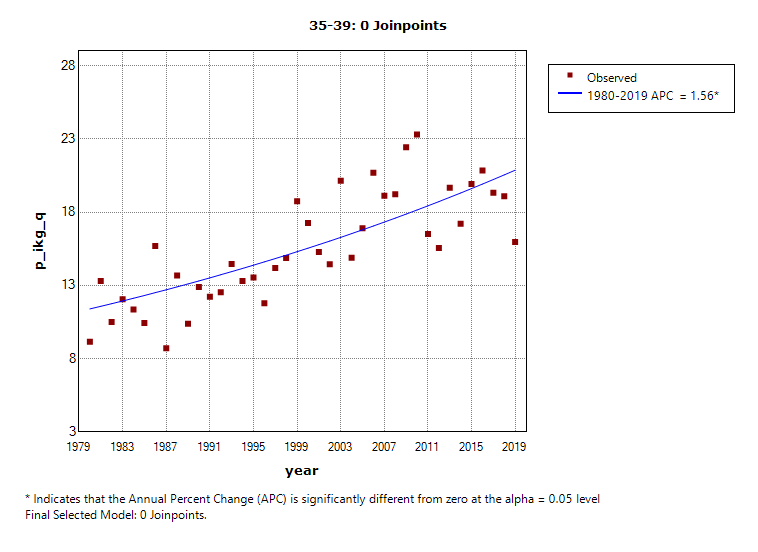 |  |

Figure S4. Trends in age-specific incidence per 100’000 person years for malignant melanoma in Switzerland between 1980 and 2019. Figure as returned from the Joinpoint software (p_ikg_q, age-specific incidence for the age groups (a) 15-19, (b) 20-24, (c) 25-29, (d) 30-34, and (e) 35-39 years).

| **(a)** |
| --- |
| 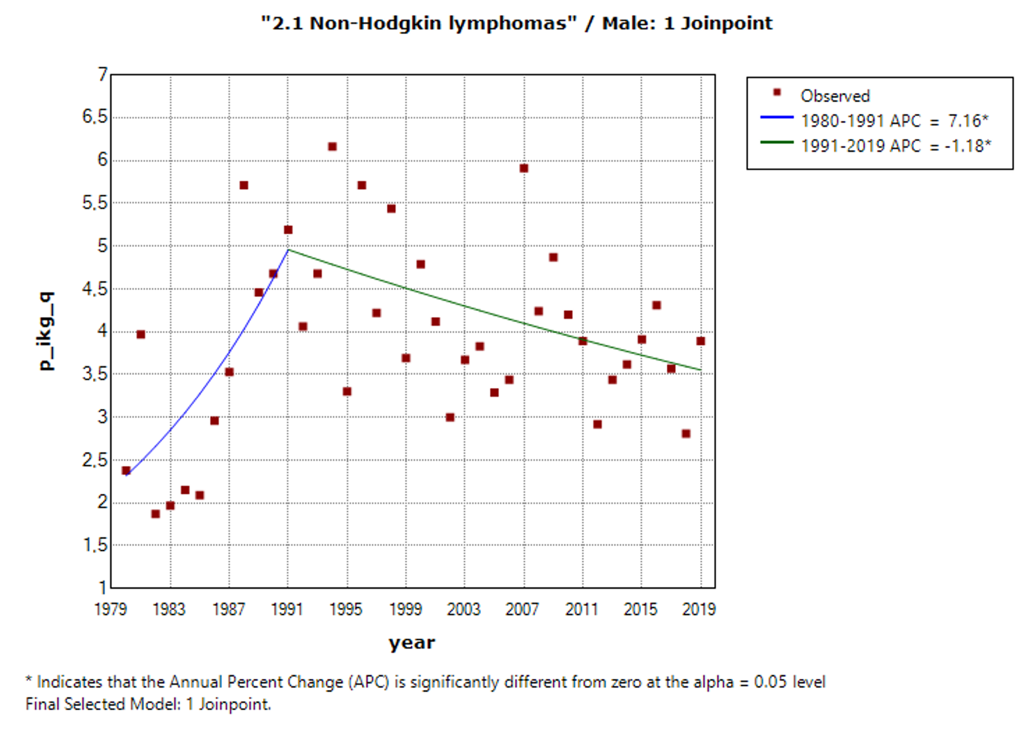 |
| (b) |
| 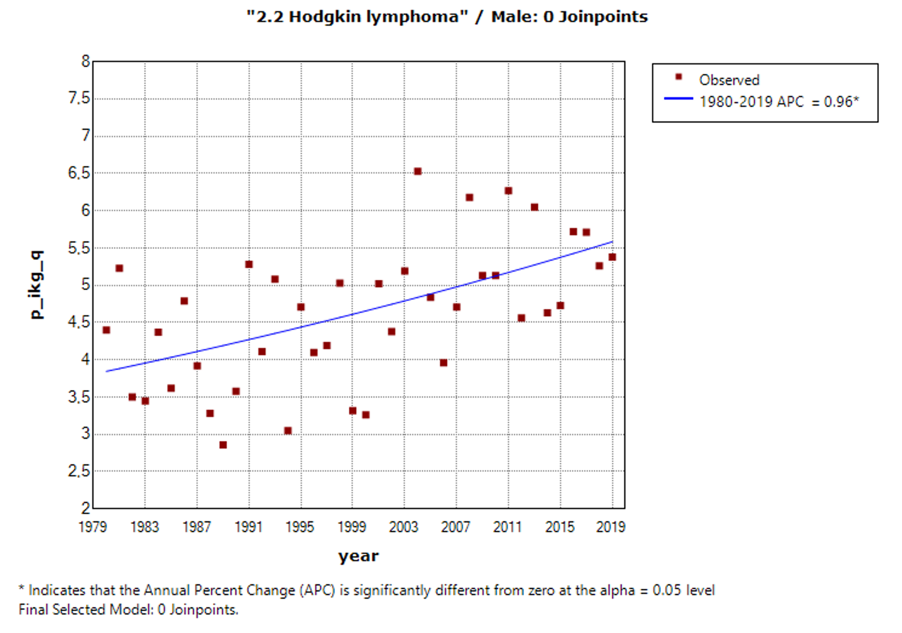 |

Figure S5. Trends in age-standardized incidence per 100’000 person years for non-Hodgkin (a) and Hodgkin lymphoma (b) in males in Switzerland between 1980 and 2019. Figure as returned from the Joinpoint software (p_ikg_q, age-standardized incidence).


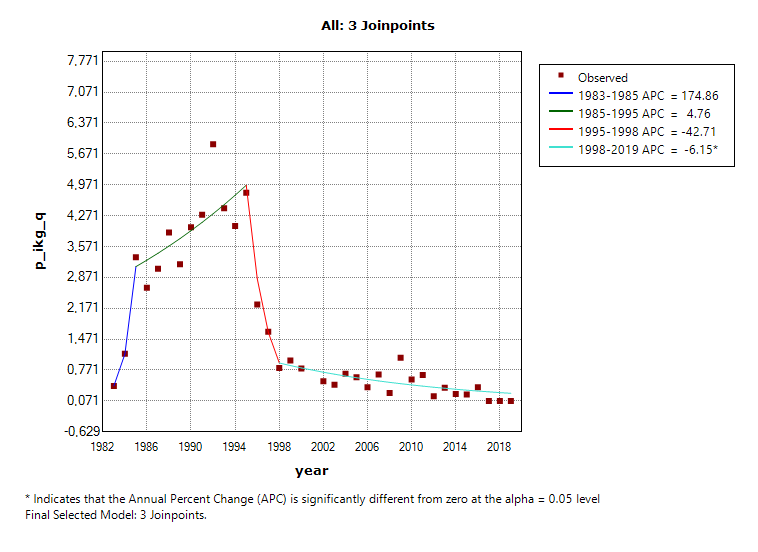


Figure S6. Trends in age-standardized incidence per 100’000 person years for Kaposi sarcoma in males in Switzerland between 1980 and 2019. Figure as returned from the Joinpoint software (p_ikg_q, age-standardized incidence).

Figure S7. Trends in age-standardized incidence per 100’000 person years for the most frequent AYA cancers (cancer category number according to Barr et al.) in females (a) and males (b) in Switzerland (solid lines - all cantons with cancer registration) and in cantons with continuous registration (dashed lines) between 1980 and 2019.

| **(a)** | **(b)** |
| --- | --- |
| **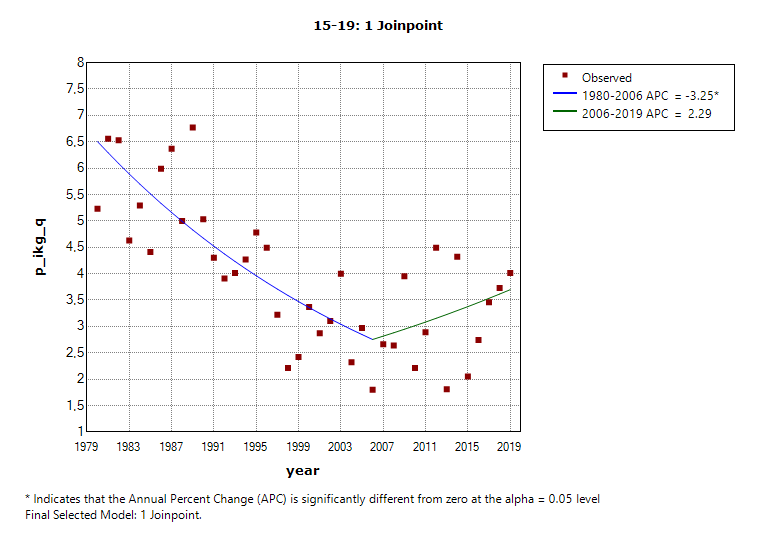** | **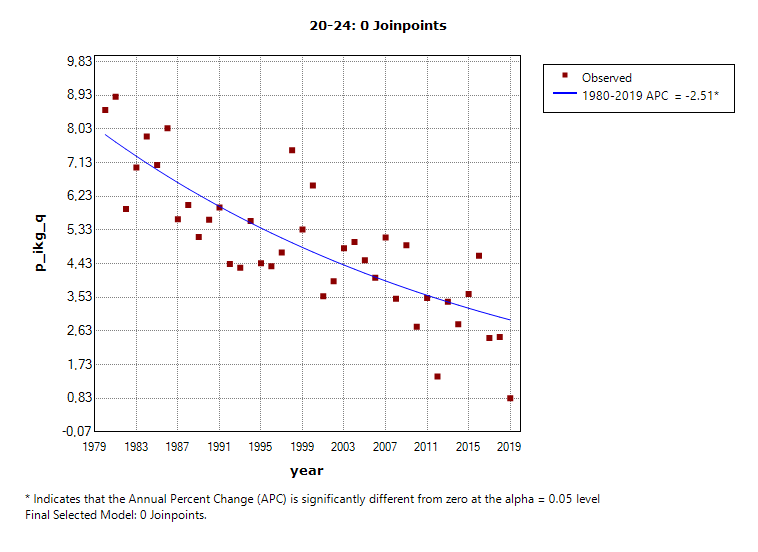** |
| **(c)** | **(d)** |
| **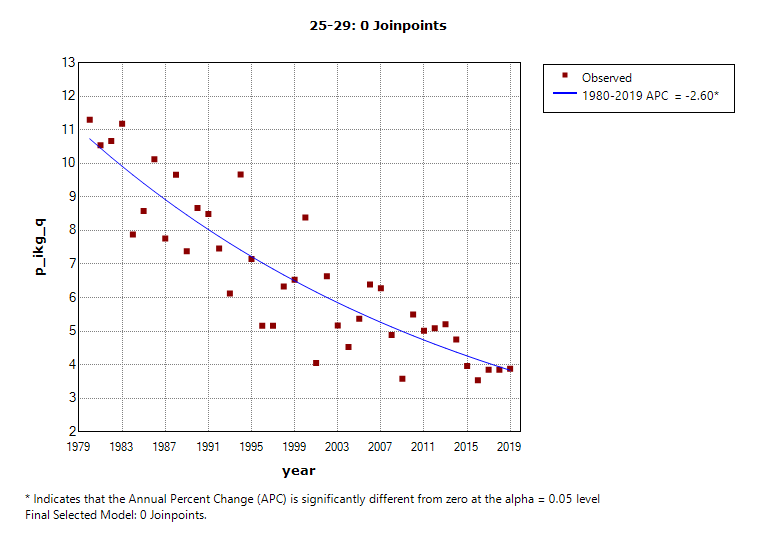** | **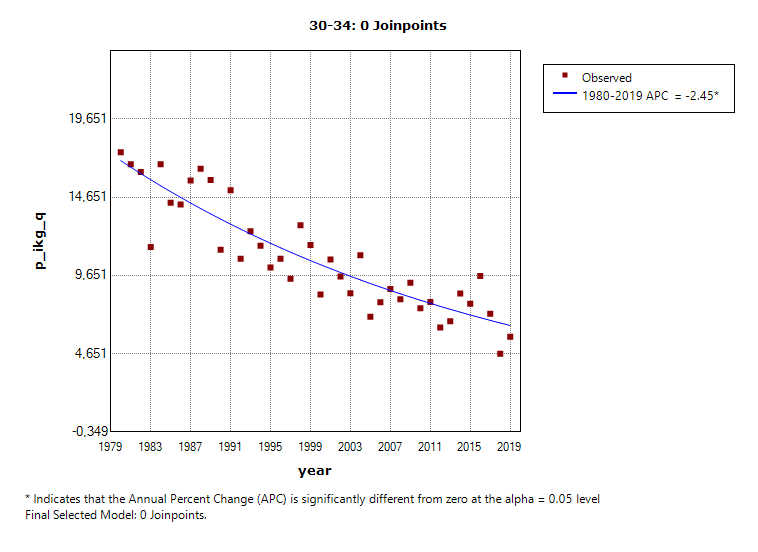** |
| **(e)** |  |
| 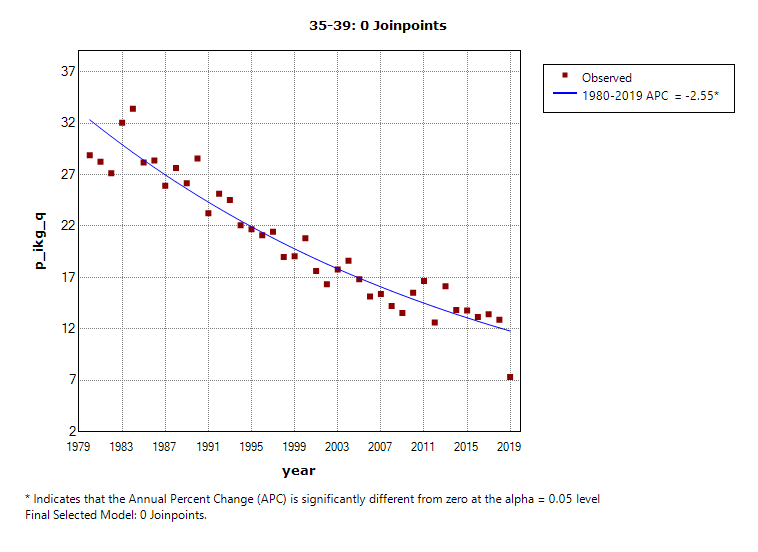 |  |

Figure S8. Trends in age-specific mortality per 100’000 person years in Switzerland between 1980 and 2019. Figure as returned from the Joinpoint software (p_ikg_q, age-specific mortality for the age groups (a) 15-19, (b) 20-24, (c) 25-29, (d) 30-34, and (e) 35-39 years).


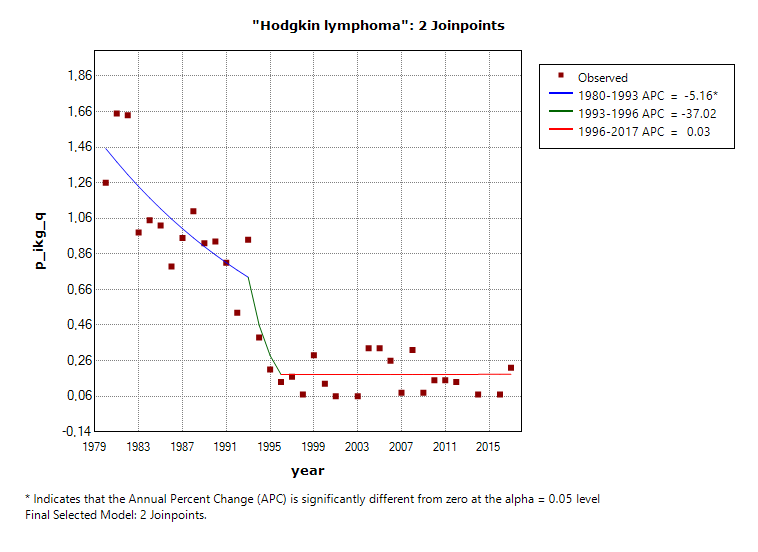


Figure S9. Trends in age-standardized mortality per 100’000 person years for Hodgkin lymphoma in males in Switzerland between 1980 and 2019. Figure as returned from the Joinpoint software (p_ikg_q, age-standardized incidence).

Figure S10. Trends in age-standardized mortality per 100’000 person years for the most frequent AYA cancers in females (a) and males (b) in Switzerland (solid lines - all cantons with cancer registration) and in cantons with continuous registration (dashed lines) between 1980 and 2019.

**References**

1. Office FS. Cantonal cancer registries: Start of data registration (Cantons) | Map [Internet]. Federal Statistical Office. 2022 [cited 2024 Aug 17]. Available from: https://www.bfs.admin.ch/asset/en/21184747

2. Barr RD, Ries LAG, Trama A, Gatta G, Steliarova‐Foucher E, Stiller CA, et al. A system for classifying cancers diagnosed in adolescents and young adults. Cancer. 2020 Nov;126(21):4634–59.
